# Primary Metabolism Is Distinctly Modulated by Plant Resistance Inducers in *Coffea arabica* Leaves Infected by *Hemileia vastatrix*

**DOI:** 10.3389/fpls.2020.00309

**Published:** 2020-03-20

**Authors:** Kátia Ferreira Possa, Joyce Alves Goulart Silva, Mário Lúcio Vilela Resende, Rita Tenente, Carla Pinheiro, Inês Chaves, Sebastien Planchon, Ana Cristina Andrade Monteiro, Jenny Renaut, Milene Alves Figueiredo Carvalho, Cândido Pinto Ricardo, Leonor Guerra-Guimarães

**Affiliations:** ^1^Departamento de Fitopatologia, Universidade Federal de Lavras, Lavras, Brazil; ^2^Centro de Investigação das Ferrugens do Cafeeiro, Instituto Superior de Agronomia, Universidade de Lisboa, Lisbon, Portugal; ^3^Instituto de Tecnologia Química e Biológica (ITQB NOVA), Universidade NOVA de Lisboa, Lisbon, Portugal; ^4^Faculdade de Ciências e Tecnologia, Universidade NOVA de Lisboa, Lisbon, Portugal; ^5^Instituto de Biologia Experimental e Tecnológica (iBET), Oeiras, Portugal; ^6^Luxembourg Institute of Science and Technology, Environmental Research and Innovation Department, Belval, Luxembourg; ^7^Embrapa Café, Parque Estação Biológica, Brasília, Brazil; ^8^Linking Landscape, Environment, Agriculture and Food, Instituto Superior de Agronomia, Universidade de Lisboa, Lisbon, Portugal

**Keywords:** coffee leaf rust (CLR), *Coffea arabica* cv. Mundo Novo, proteomics, enzymatic activities, physiological parameters, 2DE-MALDI/TOF/TOF-MS/MS, greenforce CuCa, acibenzolar-*S*-methyl (ASM)

## Abstract

Epidemics of coffee leaf rust (CLR) leads to great yield losses and huge depreciation of coffee marketing values, if no control measures are applied. Societal expectations of a more sustainable coffee production are increasingly imposing the replacement of fungicide treatments by alternative solutions. A protection strategy is to take advantage of the plant immune system by eliciting constitutive defenses. Based on such concept, plant resistance inducers (PRIs) have been developed. The Greenforce CuCa formulation, similarly to acibenzolar-*S-*methyl (ASM), shows promising results in the control of CLR (*Hemileia vastatrix*) in *Coffea arabica* cv. Mundo Novo. The molecular mechanisms of PRIs action are poorly understood. In order to contribute to its elucidation a proteomic, physiological (leaf gas-exchange) and biochemical (enzymatic) analyses were performed. Coffee leaves treated with Greenforce CuCa and ASM and inoculation with *H. vastatrix* were considered. Proteomics revealed that both PRIs lead to metabolic adjustments but, inducing distinct proteins. These proteins were related with photosynthesis, protein metabolism and stress responses. Greenforce CuCa increased photosynthesis and stomatal conductance, while ASM caused a decrease in these parameters. It was further observed that Greenforce CuCa reinforces the redox homeostasis of the leaf, while ASM seems to affect preferentially the secondary metabolism and the stress-related proteins. So, the PRIs prepare the plant to resist CLR but, inducing different defense mechanisms upon pathogen infection. The existence of a link between the primary metabolism and defense responses was evidenced. The identification of components of the plant primary metabolism, essential for plant growth and development that, simultaneously, participate in the plant defense responses can open new perspectives for plant breeding programs.

## Graphical Abstract

Main metabolic classes affected by the resistance inducers in response to coffee leaf rust.

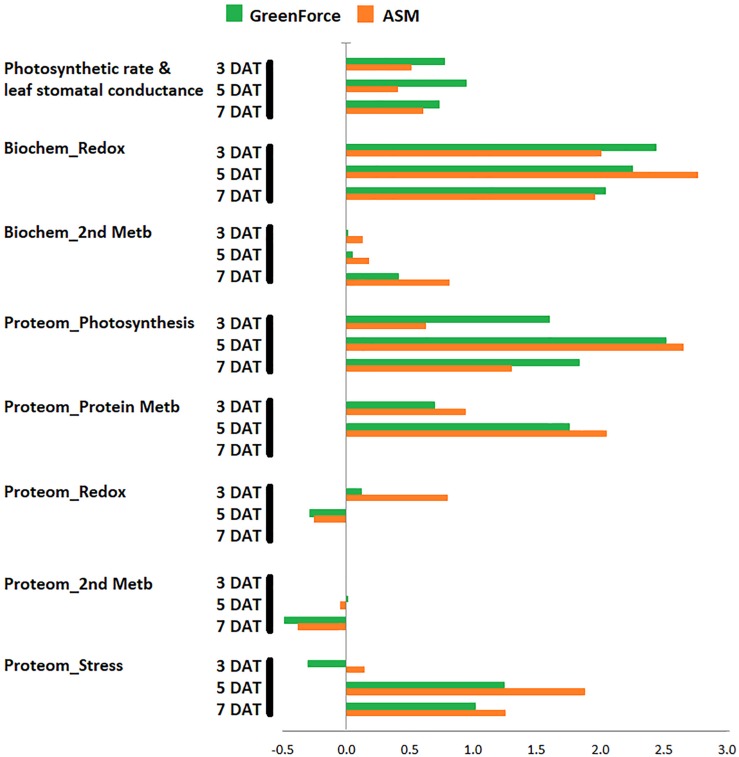

## Introduction

Coffee, one of the most important beverage crops in the world (with billions of cups consumed per day), is cultivated across Africa, Asia, and the Americas (Läderach et al., 2017). Crucial for the economy of more than 60 countries, coffee is the main source of income for more than 100 million people (Hoffmann, 2014; Läderach et al., 2017; ICO, 2020). Brazil is the first world coffee producer (mostly *Coffea arabica* L.), Minas Gerais being the state responsible for more than 50% of the Brazilian coffee production. In 2019, and considering the planted area (1.81 Mha) the productivity of coffee was 1.632 tons per hectare (CONAB, 2019).

*Coffea arabica* has the best quality/aroma, but most of its commercial varieties are highly susceptible to several pathogens, namely *Hemileia vastatrix.* This biotrophic fungus, that is spread to all coffee growing regions causes coffee leaf rust (CLR), a devastating disease characterized by large orange colonies of urediniospores in the lower surface of the leaves (Bettencourt and Rodrigues, 1988; Várzea and Marques, 2005; Talhinhas et al., 2017). In susceptible coffee leaves, after the urediniospores germination and appressorium differentiation over stomata, the fungus penetrates leaf tissues and grows into the substomatal chamber. Fungal growth continues with the formation of more intercellular hyphae and a large number of haustoria (highly specialized intracellular hyphae) in the spongy and palisade parenchyma cells. The host metabolism is modified to serve the fungus nutrients uptake allowing the completion of its life cycle (which take about 30 days) (Silva et al., 1999, 2006). The CLR causes the premature leaf fall as result of direct damages, weakening and favoring dieback of branches, decreasing the photosynthetic capacity and vigor of the infected coffee plants (Silva et al., 2006; Talhinhas et al., 2017). A recent intense epidemic of CLR in Colombia and Central America was responsible for estimated losses of several hundred million dollars (Avelino et al., 2015). In Brazil, the disease threatens coffee production, losses ranging from 30 to 50%, if no chemical control is undertaken. CLR damage is prevented by the use of protective (copper-based) and/or systemic fungicides (triazoles and strobilurin) (Zambolim, 2016). However, increasing societal expectations for sustainable coffee production demands the replacement of fungicide treatments by alternative strategies of plant protection, such as the use of coffee resistant varieties and plant resistance inducers (PRIs) (Resende et al., 2002; Várzea and Marques, 2005; Avelino et al., 2015).

The application of PRIs mimics a pathogen infection, and thus, activates a sort of unspecific systemic immunity, known as systemic acquired resistance (SAR). SAR is considered as one of the players in a multifaceted inducible defense system in plants, characterized by various signaling pathways, and metabolic responses (Cavalcanti et al., 2006; Gozzo and Faoro, 2013; Balmer et al., 2015). The PRIs application results in a stronger and faster defense response when biotic or abiotic stresses occur. Different PRI treatments, either biotic (viable or inactivated microorganisms) or chemical [e.g., acibenzolar-*S*-methyl (ASM), ethylene, plant natural formulations], have been used (Conrath et al., 2015).

Acibenzolar-*S*-methyl is a salicylic acid functional analog belonging to the benzothiadiazole (BTH) family, which is rapidly absorbed by the leaves and activating SAR (Medeiros et al., 2009; Furtado et al., 2010). Genes encoding pathogenesis-related proteins (PR proteins) are expressed and regulate secondary metabolic pathways and defense responses (Iriti and Faoro, 2003; Glazebrook, 2005; Gozzo and Faoro, 2013). ASM treatment of coffee leaves showed some protection against CLR, but without affecting *H. vastatrix* germination (Guzzo et al., 2009). Instead, what was observed was the induction by ASM of some SAR-related genes, such as those involved in: signal perception and transduction, oxidative burst and cell death, synthesis and transport of antimicrobial metabolites, synthesis of PR proteins and lipid metabolism (Guzzo et al., 2009). Under field conditions ASM application also satisfactorily controlled rust and other coffee diseases (Fernandes et al., 2013).

Formulations based on natural products have also been intensively studied, and it was found that they also activate the plant defense responses (Barguil et al., 2005; Medeiros et al., 2009; Conrath et al., 2015). Coffee industry by-products were effective in the control of *Xanthomonas vesicatoria* infection in tomato through the up-regulation of PR and antioxidant proteins (Medeiros et al., 2009). Theses formulations are being used as a control measure of plant diseases in coffee and other crops (e.g., tomato and eucalyptus) (Barguil et al., 2005; Jackson et al., 2000; Cavalcanti et al., 2006; Medeiros et al., 2009). Greenforce CuCa formulation is a plant based extract prepared with coffee industry by-products supplemented with calcium and copper salts displaying antioxidant properties. These properties derived from the high content of chlorogenic acids and caffeine, and other compounds like nicotinic acid, trigonelline, tocopherols, cafestol, and heterocyclic compounds (Esquivel and Jiménez, 2012; Murthy and Naidu, 2012). In field works, this formulation reduced CLR by about 50% (Costa et al., 2014; Silva et al., 2019).

Although the involvement of secondary metabolism in plant defense is fully documented, the relationship between defense and primary metabolism is less studied. Upon pathogen infection several genes associated with primary metabolic pathways are induced, namely, those involved in the synthesis or degradation of carbohydrates, amino acids, and lipids (Rojas et al., 2014). Links between photosynthesis and immunity in plants has been proposed (Göhre, 2015), but the role of primary metabolism in SAR has not been fully understood/analyzed. The present study aims to obtain an overview of the protein changes occurring in the *C. arabica* cv. Mundo Novo leaves upon treatment with the resistance inducers Greenforce Cuca and ASM and, subsequently, challenged by the obligate biotrophic fungus *H. vastatrix*. This proteomic phenotyping was complemented by physiological and biochemical analyses (leaf gas-exchange and enzymatic assays).

## Materials and Methods

### Biological Material and Treatments

Six month old seedlings (with five leaf pairs) of *C. arabica* cv. Mundo Novo IAC 376/4 (susceptible to CLR) were used. The experiment was conducted in a growth chamber (Eletrolab) at 24°C and 12 h photoperiod with fluorescent light (600 μmoles m^–2^ s^–1^). Plants were acclimated in the growth chamber, 30 days prior to the beginning of the experiment.

The upper surface of young fully expanded leaves of *C. arabica* cv. Mundo Novo were sprayed with two resistance inducers (PRIs), ASM (Bion 500 WG, Syngenta) and Greenforce CuCa (formulation prepared from products of coffee industry supplemented with copper and calcium salts; Universidade Federal de Lavras – patent pending PI063575-2, National Institute of Industrial Property, Brazil). The doses of the products were set according to the manufacturer’s recommendations: 0.2 g L^–1^ for ASM and 5 mL L^–1^ for Greenforce CuCa. Approximately 1 mL of the PRI solutions per leaf, using a manual sprayer was used. Leaves sprayed with water were used as mock-treated control.

**FIGURE 1:**
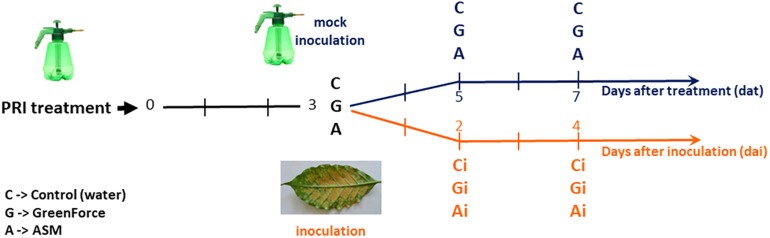
Experimental design – *Coffea arabica* cv. Mundo Novo leaves were either treated with the PRIs, ASM (A) and Greenforce CuCa (G) or subsequently inoculated with *Hemileia vastatrix* (Ai, Gi). Leaves were collected at 3, 5, and 7 days after PRIs treatment (black timeline) and at 2 and 4 days after *H. vastatrix* inoculation (orange timeline). Leaves treated with water were used as control: mock-treated/inoculated (C) or inoculated (Ci).

Urediniospores of *H. vastatrix* obtained from diseased field grown coffee (*C. arabica* cv. Mundo Novo) were used as inoculum source. Inoculation was performed at 3 days after PRIs treatment by spraying the undersurfaces of the leaves with a urediniospore suspension in 0.2% agar distilled water (v/v) with 0.05% Tween to a final concentration of 10^6^ urediniospore mL^–1^. After inoculation all plants were kept for 24 h in a dark moist chamber (Guzzo et al., 2009). Leaves sprayed with water and kept in the same conditions of the inoculated leaves were used as mocked-inoculated control. Samples were collected at 3, 5, and 7 days after PRIs treatment, which correspond to 0, 2, and 4 days after *H. vastatrix* inoculation, respectively (Figure 1).

A complete randomized block design with six treatments and three blocks (replicates) per time-point was performed. Three plants (one leaf pair per plant) were used as experimental unit for either proteomic, physiological or biochemical studies.

The rust disease severity assessment (percentage of affected area) was done by visual evaluation of the inoculated leaves (four leaves for plant) after the appearance of the first symptoms in a total of six evaluations (every 7 days) according to a diagrammatic scale (Capucho et al., 2011). The observed indexes of severity were transformed in the area under the corresponding disease severity progress curve (AUDsPC) as proposed by Shaner and Finney (1977) (Supplementary Tables S1A,B).

### Proteomic Analysis

Coffee leaves (1 g) were ground with liquid nitrogen and proteins extracted using the trichloroacetic acid (TCA) precipitation method (Damerval et al., 1986) and recovered in 30 mM Tris-HCl pH 8.8 buffer solution containing 2% SDS and 50 mM DTT. After 2 h of agitation the samples were centrifuged at 12000 *g*, during 15 min and the clear supernatants were purified using the 2D clean-up kit following the manufacturer instructions (GE Healthcare). The precipitates were resuspended in 30 mM Tris-HCl pH 8.5 buffer containing 7M urea, 2M thiourea and 4% CHAPS; protein content was measured using a modified Bradford assay method (Ramagli, 1999).

Protein samples (300 μg) were run in 18 cm long IPG strips, pH 4–7 L (GE Healthcare). IEF was performed using the Ettan IPGphor (GE Healthcare) under the following conditions: a total of 32000 Vhrs at 20°C; Step-n-hold 100 V–2 h; Step-n-hold 30 V–10 h; Step-n-hold 150 V–3 h; Step-n-hold 300 V–3 h; Gradient 1000 V–6 h; Step-n-hold 1000 V–1 h; Gradient 8000 V–4 h; Step-n-hold 8000 V–3200 V/h; Step-n-hold 100 V–18 h; maximum current setting of 50 μA per strip. After IEF, the proteins in the IPG strip were equilibrated for 15 min in a buffer (100 mM Tris–HCl pH 8.8, 6M urea, 2% SDS, 30% glycerol and 0.2 mg mL^–1^ bromophenol blue) containing 5 mg mL^–1^ DTT (to reduce proteins), followed by another 15 min equilibration in the same buffer but which had DTT replaced by 25 mg mL^–1^ iodoacetamide (to alkylate proteins). The second dimension SDS-PAGE was performed at 25°C with 12% resolving gels using the 2-D Ettan Dalt II Gel apparatus (GE Healthcare) at 10 mA for 15 min and then at 20 mA until the bromophenol blue dye front had run off the gel (around 22 h). The molecular mass markers used were the “Precision Plus Protein All Blue Standards” (Bio-Rad, Hercules, CA, United States).

Gels were stained in Colloidal Coomassie Blue (Neuhoff et al., 1985) and the protein profiles were scanned using an ImageScanner II (Amersham Biosciences). The image gel analysis was carried out using the Progenesis SameSpots 2D software v. 4.5 (Non-linear Dynamics, Ltd.). The spot volumes were normalized using the mean value of the replicates (Grove et al., 2008). One-way ANOVA analyses were performed using a *p*-value of 0.05. For the proteins with statistically significant changes (and a fold change > 1.5) a principal component analysis (PCA) was carried out and a hierarchical clustering was performed applying a Pearson correlation using the MeV5 v. 4.8 (Supplementary Table S2).

Polypeptide spots visually detected in Colloidal Coomassie Blue stained gels were excised from the gels and processed using the Tecan freedom EVO200 (Tecan, Männedorf, CH) as previously described (Guerra-Guimarães et al., 2015). The ProteinPilot^TM^ software 4.0.8085 was used for database searches with an in-house MASCOT platform (version 2.3, Matrix Science^1^, London, United Kingdom). All proteins were identified by search against a *Coffea* database downloaded from NCBI^2^ on January 17, 2019 with the taxID 13442 and containing 133 773 sequences. All searches (combined MS and 10 MS/MS spectra) were carried out using a mass window of 100 ppm for the precursor and 0.5 Da for the fragments. During the different searches the following parameters were defined: two missed cleavages, carbamidomethylation of cysteine as fixed modification, and as variable modifications we selected; oxidation of methionine, single or double oxidation of tryptophan and tryptophan to kynurenine. The proteins identified without clear annotation were BLAST analyzed and those proteins with the highest homology (when significant) were added to the Supplementary Table S3.

All identifications were manually validated and extra precursors were selected for fragmentation if the obtained data were judged as insufficient. When high quality spectra were not matched to sequences, a sequence was determined manually and, so, in the current data set they could be linked to the identified protein by allowing for more missed cleavages, semi-tryptic peptides, specific modifications or broader taxonomy search. All the spots identified in the 5 day sample control, containing one or more proteins were considered for the leaf proteome establishment. For the comparative analysis only spots having unique and significant protein identification were considered.

The mass spectrometry proteomics data have been deposited to the ProteomeXchange Consortium (Deutsch et al., 2017) via the PRIDE partner repository (Perez-Riverol et al., 2019) with the dataset identifier PXD016012.

The identified proteins were also subjected to InterPro, UniProt, and NCBI databases analyses. The conserved domains of each protein, as well as, the superfamily were determined using the NCBI tools^2^. Assignment for functional annotation and protein location was based on MapMan ‘Bin’ ontology^3^ using Mercator Automated Sequence Annotation Pipeline^4^ (Lohse et al., 2014) and on Gene Ontology Annotation (GO^5^) using Blast2GO software (version 5 basic^6^) (Conesa and Götz, 2008). In addition, protein subcellular location was also assigned using the LocTree3^7^ (Goldberg et al., 2014). Default parameters were used for all the programs.

### Physiological Measurements

Gas-exchange characteristics were evaluated in fully expanded leaves using a LI-6400XT Portable Photosynthesis System (LI-COR, Lincoln, United Kingdom), at a photosynthetic photon flux density (PPFD) of 1000 μmol m^–2^ s^–1^, from a red/blue light source (6400-02B LI-COR, Lincoln, United Kingdom LED). Measurements took place between 8:30 and 11:30 h (solar time). Two leaves per plant (3^*rd*^ leaf pair), in a total of six leaves were used per experimental unit. Leaf net photosynthetic rate (*A*; μmol CO_2_ m^–2^ s^–1^), stomatal conductance (*g*_*s*_; mol H_2_O m^–2^ s^–1^), mesophyll intercellular CO_2_ concentration (*c*_*i*_), the ratio of intercellular to ambient CO_2_ concentrations (*c_*i*__/_c_*a*_*), water use efficiency (WUE, *A*/*transpiration*) and carboxylation efficiency of photosynthesis (*A/c_*i*_*) were estimated. Data related to each time-point was analyzed by one-way analysis of variance (ANOVA). When treatments were significant by the *F*-test, a pairwise multiple comparison was performed using Tukey test (*p* ≤ 0.05).

### Biochemical Assays

The activities of peroxidase (POX), ascorbate peroxidase (APX), superoxide dismutase (SOD), phenylalanine ammonia lyase (PAL), and polyphenol oxidase (PPO) were quantified. Protein content was determined using bovine serum albumin (BSA) as standard (Bradford, 1976). Data related to each time-point was analyzed by one-way analysis of variance (ANOVA). When treatments were significant by the *F*-test, a pairwise multiple comparison was performed using Tukey test (*p* ≤ 0.05).

POX, APX, and SOD measurements were made according to Biemelt et al. (1998) with some modifications. Leaves (0.2 mg) were ground with liquid nitrogen in the presence of 1% polyvinylpolypyrrolidone (PVPP) (w/w) and extracted with 1.5 ml of potassium phosphate buffer (100 mM, pH 7.8) containing 0.1 mM ethylenedinitrilotetraacetic acid (EDTA) and 10 mM ascorbic acid. The extract was centrifuged at 13,000 *g*, 4°C for 15 min and supernatants used for the enzymatic analyses.

POX activity was performed according to Urbanek et al. (1991) at 30°C, and recording the absorbance at 480 nm. POX specific activity (μmol/min/mg protein) was calculated using the molar absorption coefficient (**ε**) of 1.235 mM^–1^ cm^–1^ (Chance and Maehly, 1955).

APX activity was performed according to Nakano and Asada (1981) at 25°C, and recording the absorbance at 290 nm. APX specific activity (μmol/min/mg protein) was calculated using the molar absorption coefficient (**ε**) of 1.4 mM^–1^ cm^–1^.

SOD activity was performed according to Giannopolitis and Ries (1977) following the nitro blue tetrazolium chloride (NBT) photoreduction, by measuring the absorbance at 560 nm. Data was presented as SOD specific activity (U/min/mg protein). One unit of SOD activity was defined as the amount of enzyme able to reduce NBT photoreduction by 50%.

For PAL quantification, and after liquid nitrogen grinding, 1 g of leaves were extracted with 3 mL of sodium phosphate buffer (50 mM, pH 6.5) containing 0.1 mM phenylmethylsulfonyl fluoride (PMSF) and 1% PVPP (w/v). The extract was centrifuged at 13,000 *g*, 4°C for 25 min, using the supernatant for enzymatic analysis. PAL activity was performed according to Zucker (1965) at 37°C, and recording absorbance at 280 nm. PAL specific activity (μmol/min/mg protein) was calculated by the phenylalanine decrease using the molar absorption coefficient (**ε**) of 10000 mM^–1^ cm^–1^.

For PPO quantification, after liquid nitrogen grinding, 1 g of leaves were extracted with 4 mL of potassium phosphate buffer (30 mM, pH 7.0) containing 0.1 mM EDTA. The extract was centrifuged at 13,000 *g*, 4°C for 25 min and using the supernatant for enzymatic analysis. PPO activity was performed according to Kar and Mishra (1976) at 30°C, recording absorbance at 410 nm. PPO specific activity (μmol/min/mg protein) was calculated through catechol degradation using the molar absorption coefficient (**ε**) of 1.235 mM^–1^ cm^–1^.

Hydrogen peroxide (H_2_O_2_) content was estimated in 0.2 g of leaves ground in liquid nitrogen, according to Velikova et al. (2000). The extract was centrifuged (12000 *g*, 4°C for 15 min), and the supernatant was used for H_2_O_2_ estimation at 390 nm, using a standard curve prepared with the following H_2_O_2_ concentrations: 0, 5, 15, 25, 35 and 45 μmol/ml.

Lipid peroxidation was determined in 0.2 g of leaves ground in liquid nitrogen, according to Buege and Aust (1978). The extract was centrifuged (10,000 *g*, 4°C for 10 min) and the supernatant was incubated at 95°C for 30 min. The reaction was stopped by lowering the temperature 0°C. The absorbance was recorded at 535 and 600 nm and the malondialdehyde (MDA) content was calculated through the formula: [MDA] = (A535–A600) using the molar absorption coefficient (ε) of 1.56 × 10^–5^ mM^–1^ cm^–1^.

## Results

### Coffee Leaf Proteome

Three hundred and fifty polypeptide spots were detected by 2-DE (linear pH gradient of 4–7) in extracts of the control water-treated leaves. Proteins could be successfully identified in 179 of these spots, 43 of them containing more than one protein (Supplementary Figure S1). All the protein identifications were achieved within the *Coffea* genomes, mainly *C. arabica* (107 proteins) and *C. eugenioides* (97 proteins). The 219 identified proteins belong to 66 superfamilies according to their conserved domains (Supplementary Table S4). The five most represented superfamilies were: FliI (25 spots); RuBisCO_large (14 spots), P-loop_NTPase (13 spots); GH18-Chitinase (9 spots) and Chloroa_b-bind superfamily (7 spots).

The 25 FliI superfamily spots are ATP synthases, one is a mitochondrial ATP synthase related with oxidative phosphorylation; five are V-ATPases related with transport across tonoplast; and nine are ATP synthase CF1 related with photochemical reactions. Also related with the photochemical pathway are the 10 chlorophyll a-b binding proteins from the Chloro a_b-bind and PLN00048 superfamilies (photosystem light harvesting chlorophyll a/b binding proteins from both LHCI and LHCII). Largely represented is the RuBisCO_large subunit superfamily (14 spots), but RuBisCO related proteins represent a much larger proportion since RuBisCO activase (P-loop_NTPase superfamily, 13 spots), RuBisCO small subunit (4 spots) and RuBisCO chaperonins (Chaperonin_like superfamily, 7 spots) were also detected. Glycosyl hydrolases were also found, namely acidic endochitinase-like proteins (GH18-Chitinase superfamily, 9 spots) and one alpha-mannosidase (GH38-57_N_LamB_YdjC_SF superfamily).

**FIGURE 2:**
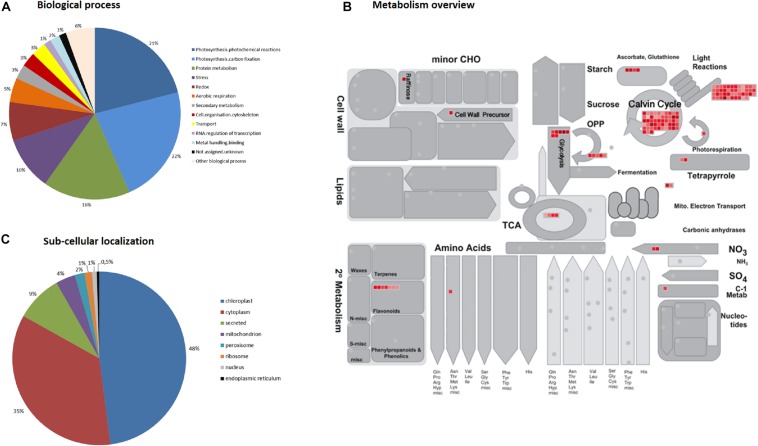
Functional annotation of the 219 identified coffee leaf proteins based on MapMan “Bin” and GO ontology. **(A)** Biological process; **(B)** Metabolism overview; **(C)** Sub-cellular localization.

Functional categorization of the 219 identified proteins, based on MapMan “Bin” and GO annotation, indicated that they are mostly involved in: photosynthesis (43%), protein metabolism (16%), stress (10%), redox (7%), aerobic respiration (5%), and secondary metabolism (3%) (Figures 2A,B). Their predicted localizations are: chloroplast (48%), cytoplasm (35%), mitochondrion (4%), peroxisome (2%), ribosome (1%), nucleus (1%), endoplasmic reticulum (0.5%), and secreted (9%) (Figure 2C). Considering that for 124 out of the 219 proteins (56%) EC numbers were assigned (Blast2GO analysis), it was found that 53 spots were hydrolases (ATPases and NTPases), 26 were oxidoreductases, 26 were lyases (carboxy-lyases) and 11 were transferases (Supplementary Table S4). Blast2GO did not assign EC numbers to nine spots identified as acidic endochitinase-like proteins, but they will eventually have hydrolytic activity. Furthermore, distinct isoforms of the same protein (with different pI) were detected for several spots; such as: sedoheptulose-bisphosphatase (spots #853, #857), RuBisCO activase (spots #759, #756), phosphoglycerate kinase (spots #684, #688), RuBisCO large subunit (spots #628, #624) and Glyceraldehyde-3-phosphate dehydrogenase (spots #699, #700, #1500).

### Changes in the Leaf Proteome Due to PRI Treatments and *H. vastatrix* Infection

**FIGURE 3:**
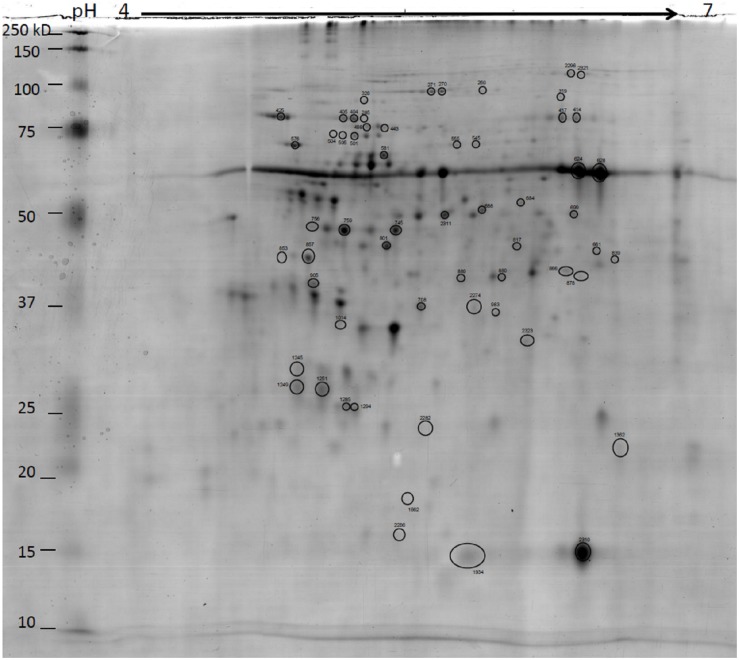
Representative 2DE gels of coffee leaf proteins. Circled spots that significantly changed in abundance between Greenforce CuCa (G), ASM (A) and Control (C) at 3, 5, and 7 dat and 2 and 4 days after inoculation (i) with *H. vastatrix* (Hv) [one way ANOVA analysis was performed using a *p-*value < 0.05 and Fold Change > 1.5.] The proteins were successfully identified by MALDI-TOF/TOF-MS (detailed information on Table 2). Gels were stained in Colloidal Coomassie Blue.

The 2-DE protein patterns of coffee leaves treated with the PRIs (Greenforce CuCa and ASM) at 3, 5 and 7 days were analyzed. The simultaneous effect of PRI treatments and *H. vastatrix* infection at 2 and 4 days after inoculation (dai) were also studied. Overall, the number of spots that changed in abundance for all the comparisons were 165, of which 112 have an unique protein identification and were, thus, considered for further analysis (Table 1). These 112 spots represent 58 distinct proteins, since some proteins were present in more than one sample/comparison/time-point (Table 2 and Figure 3). These 58 proteins represent 26% of the 219 reproducibly identified proteins of the leaf proteome shown in Supplementary Table S4.

**TABLE 1:** Proteomic analysis of coffee leaves treated with PRIs and infected with *Hemileia vastatrix*.

Assessment	Comparisons	Days	Number of spots*	Spots with unique ID
PRI treatments	C x G x A	3	16	12
		5	44	24
		7	13	9
PRI + Hv treatments	Ci x Gi x Ai	5 (2)	20	14
		7 (4)	13	8
Multiple interactions	C x G x A x Ci x Gi x Ai	5 (2)	44	34
		7 (4)	15	11

Number of spots that significantly changed in abundance due to PRI treatments Greenforce CuCa (G) and ASM (A) and Control leaves (C) at 3, 5, and 7 days after treatments, and at 2 and 4 days after inoculation (i) with H. vastatrix (Hv).*ANOVA (p-value < 0.05) and Fold Change > 1.5.

**TABLE 2:** Annotation of the coffee leaf proteins that changed in abundance after PRI treatments and *H. vastatrix* infection.

Biological process^a^	Spot no.^b^	Protein identification^c^	Accession no.^d^	Days^e^
Aerobic respiration	555	2,3-Bisphosphoglycerate-independent phosphoglycerate mutase	1531848506	5
	2323	Triosephosphate isomerase, cytosolic	1527522406	5
	319	NADH dehydrogenase [ubiquinone] iron-sulfur protein 1, mitochondrial	1527604080	3
	2321	Aconitate hydratase, cytoplasmic	1527562521	5
C1-metabolism	2274	*S*-Formylglutathione hydrolase-like isoform X1	1527584126	5
Hormone metabolism	878	Auxin-induced protein PCNT115-like	1531837410	5
Minor CHO metabolism	260	Galactinol–sucrose galactosyltransferase	1531812739	5
Misc.enzymes.oxidases	817	2-Methylene-furan-3-one reductase-like	1527480760	7
N-metabolism	2311	Glutamine synthetase leaf isozyme, chloroplastic	1531836679	5
Photosynthesis	414	Transketolase, chloroplastic	1527513560	5
	417	Transketolase, chloroplastic	1531823890	5,7
	699	Glyceraldehyde-3-phosphate dehydrogenase B, chloroplastic	1527478725	3,5,7
	684	Phosphoglycerate kinase, chloroplastic	1527563431	5
	688	Phosphoglycerate kinase, chloroplastic	1527557669	5
	801	Phosphoribulokinase, chloroplastic-like	1527610096	5
	745	Ribulose bisphosphate carboxylase/oxygenase activase, chloroplastic-like isoform X2	1527509480	7
	756	Ribulose bisphosphate carboxylase/oxygenase activase, chloroplastic-like isoform X2	1531823432	3, 5
	759	Ribulose bisphosphate carboxylase/oxygenase activase, chloroplastic-like isoform X2	1531823432	5
	545, 624	Ribulose-1,5-bisphosphate carboxylase/oxygenase large subunit, partial (chloroplast)	11230404	7
	628	Ribulose-1,5-bisphosphate carboxylase/oxygenase large subunit (plastid)	1184802834	5,7
	1934, 2310	Plastid ribulose-1,5-bisphosphate carboxylase/oxygenase small subunit, partial	83375926	5
	853, 857	Sedoheptulose-1,7-bisphosphatase, chloroplastic	1527522116	5
	501	ATP synthase CF1 alpha subunit (plastid)	1184802828	5,7
	270	ATP synthase CF1 alpha subunit (plastid)	1184802831	5
	866	ATP synthase gamma chain, chloroplastic	1527619509	3, 5
	1285	Chlorophyll a-b binding protein 8, chloroplastic	1527556260	5
	1362	Photosystem I reaction center subunit II, chloroplastic-like	1531835629	5
	1245	Chlorophyll a-b binding protein of LHCII type 1	1531876544	7
	1249	Chlorophyll a-b binding protein of LHCII type 1-like	1527620709	3
	1251	Chlorophyll a-b binding protein of LHCII type 1-like	1531879126	3
	1294	Chlorophyll a-b binding protein 36, chloroplastic	1531849907	3, 5
	1014	Oxygen-evolving enhancer protein 1, chloroplastic	1531816163	5
	2282	Oxygen-evolving enhancer protein 2, chloroplastic-like	1531849935	5, 7
Protein.degradation	498, 504	ATP-dependent zinc metalloprotease FTSH 2, chloroplastic	1531812697	5
	505	ATP-dependent zinc metalloprotease FTSH 2, chloroplastic	1531812697	3,5
	271	ATP-dependent Clp protease ATP-binding subunit ClpA homolog CD4B, chloroplastic	1531841929	5
Protein.folding	576	LOW QUALITY PROTEIN: RuBisCO large subunit-binding protein subunit alpha-like	1527493650	5
	581	RuBisCO large subunit-binding protein subunit beta, chloroplastic	1531799225	3,5
Protein.synthesis	328	Elongation factor G-2, chloroplastic-like	1527609809	5
	2296	Elongation factor 2	1531868107	3
Redox	983	L-Ascorbate peroxidase, cytosolic-like	1527502141	5
	1662	Thioredoxin H-type-like	1527479082	3
	2286	Thioredoxin M4, chloroplastic-like	1531852444	7
RNA	661	Chloroplast stem-loop binding protein of 41 kDa a, chloroplastic-like	1527504431	7
Secondary metabolism	880	Isoflavone reductase homolog PCBER-like	1531818231	5
	889	Isoflavone reductase homolog PCBER-like	1531818231	7
Stress	285	Heat shock cognate 70 kDa protein 2-like	1531823375	7
	404	Heat shock cognate 70 kDa protein 2	1531799105	3, 5
	405	Heat shock cognate 70 kDa protein 2	1531799105	5,7
	425	Stromal 70 kDa heat shock-related protein, chloroplastic-like	1527478027	5
	443	Heat shock 70 kDa protein, mitochondrial	1527512911	5,7
	839	Auxin-binding protein ABP20-like	1527581463	5, 7
	905	Acidic endochitinase-like	1527489669	5
Unknown.not assigned	708	Abscisic stress-ripening protein 5-like	1531806318	7

*^a^Functional characterization of the proteins based on MapMan ‘Bin’ and Go ontology.^b^The number that identified protein spots on 2-DE gel (Figure 3).^c^The proteins were identified by MALDI-TOF/TOF-MS/MS followed by homology search in Coffea NCBI database.^d^The accession number from GenBank assigned to the polypeptide after MS/MS analysis.^e^Days after PRIs treatments.*

**FIGURE 4:**
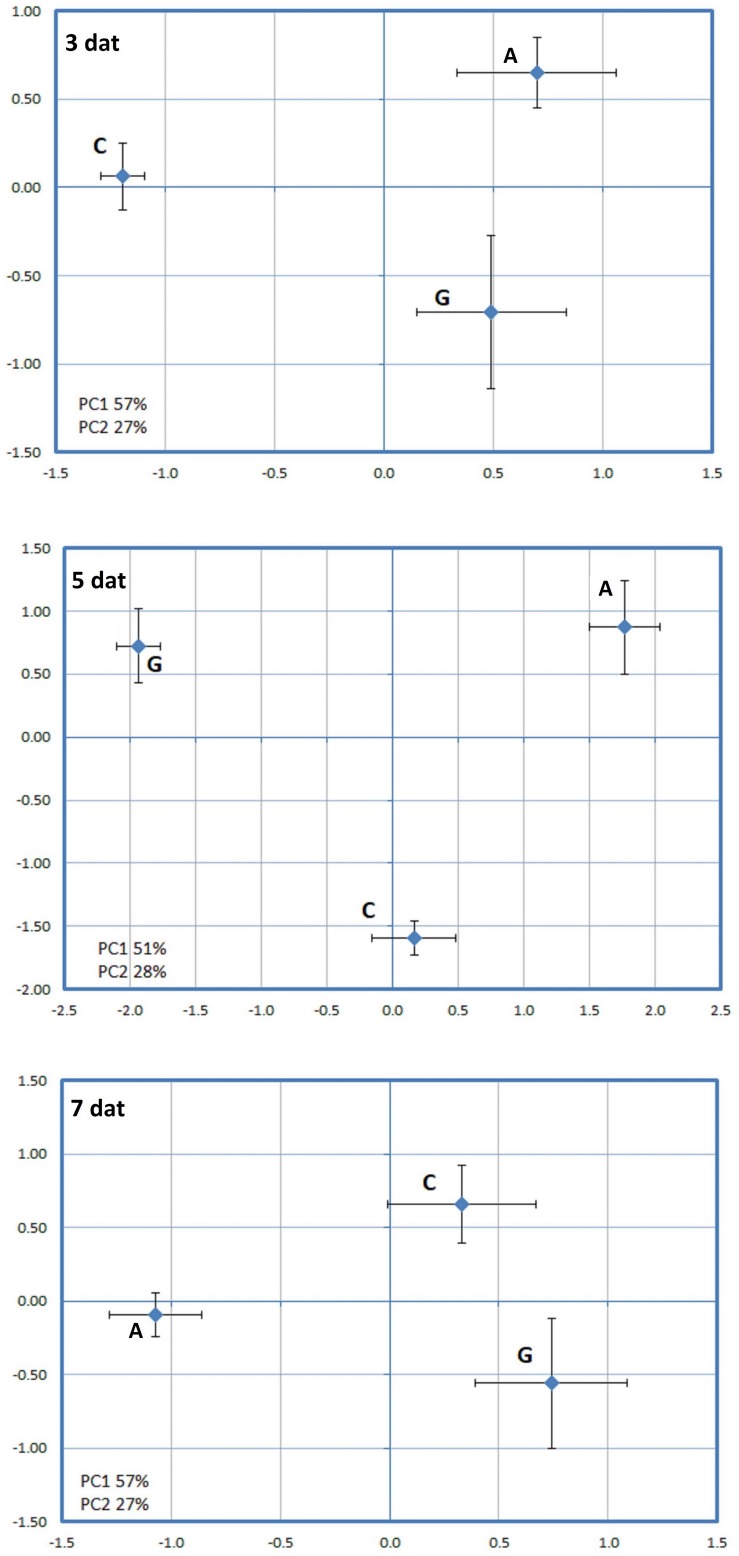
Principal component analysis (PCA) performed for the spots whose volume significantly changed in abundance (*p*-value < 0.05) between Greenforce CuCa (G), ASM (A), and Control (C) at 3, 5 and 7 days after treatments.

**FIGURE 5:**
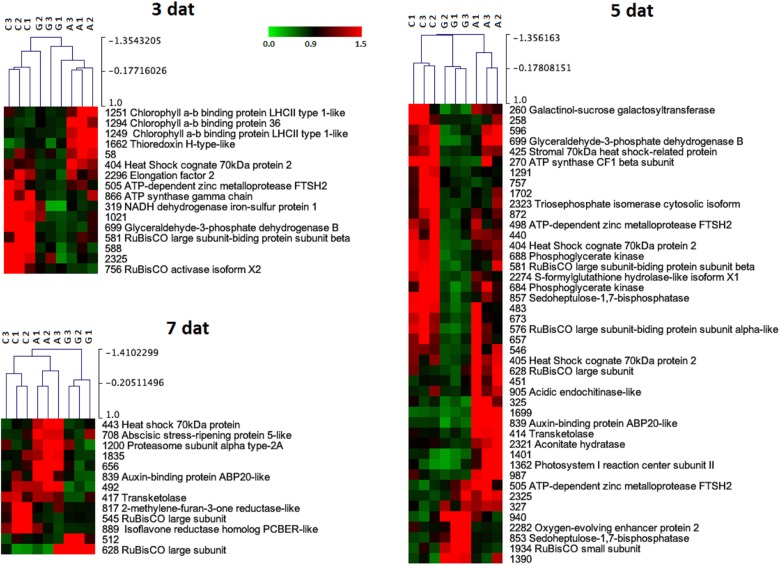
Hierarchical cluster analysis of the proteins that significantly changed in abundance (*p*-value < 0.05) between Greenforce CuCa (G), ASM (A), and Control (C) at 3, 5, and 7 days after treatments. The signals are shown in a red-green color scale, from a gradient of red (higher expression) to green (lower expression).

A principal components analysis (PCA) applied to the proteins that changed in abundance due to the PRI treatments [Greenforce CuCa (G), ASM (A), and Control (C)] at 3, 5, and 7 days (Figure 4) revealed a clear separation of the samples. The hierarchical cluster analysis additionally showed the variation in abundance of the proteins in the samples (Figure 5). At 3 days after the treatments (dat), 16 spots significantly differed between samples. This number increased to 44 spots at 5dat and decreased to 13 spots at 7dat. Therefore, at day 5 the protein profiles were more diverse.

**FIGURE 6:**
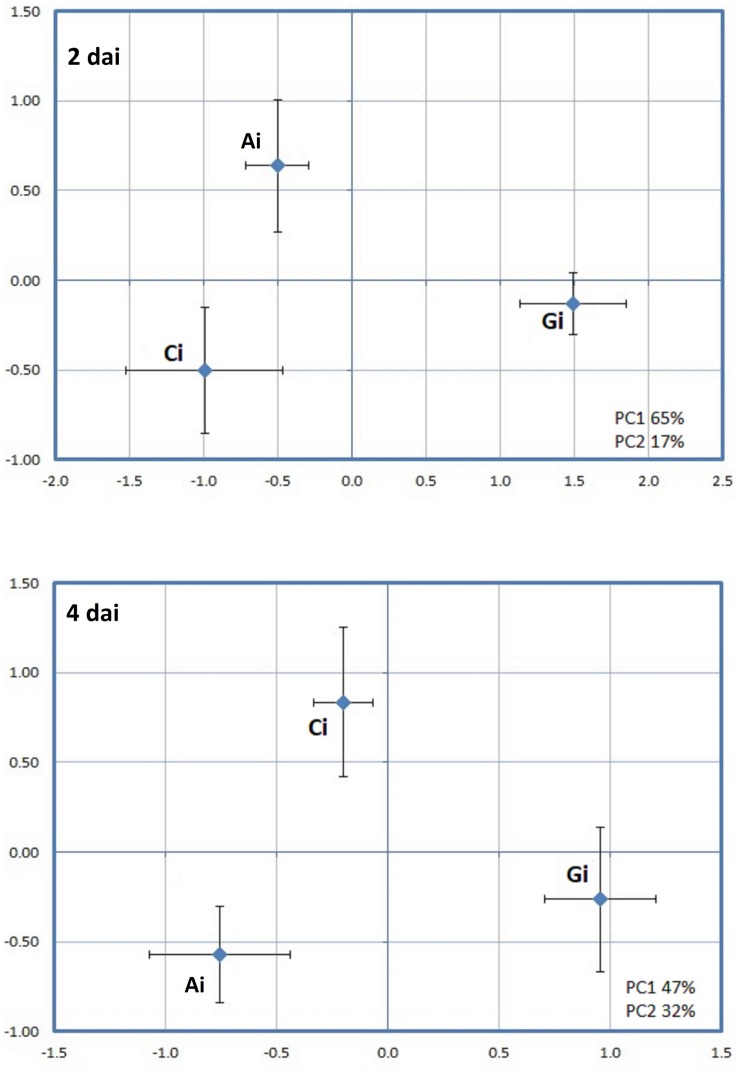
Principal component analysis (PCA) performed for the spots whose volume significantly changed in abundance (*p*-value < 0.05) between PRI treatments followed by *H. vastatrix* infection, at 2 and 4 days after inoculation (i): Greenforce CuCa (Gi), ASM (Ai), and Control (Ci).

**FIGURE 7:**
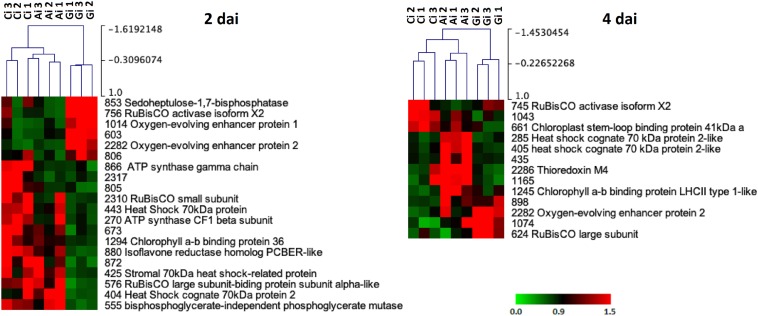
Hierarchical cluster analysis of the proteins that significantly changed in abundance (*p*-value < 0.05) between PRI treatments followed by *H. vastatrix* infection, at 2 and 4 days after inoculation (i): Greenforce CuCa (Gi), ASM (Ai), and Control (Ci). The signals are shown in a red-green color scale, from a gradient of red (higher expression) to green (lower expression).

When a PCA was applied to the proteins that were effect by PRI treatments followed by the *H. vastatrix* infection at 2 or 4 days (Figure 6), it was also observed a clear separation of the three conditions [Greenforce CuCa infected (Gi), ASM infected (Ai), and Control infected (Ci)]. The hierarchical cluster analysis showed the variation of protein abundance in the samples (Figure 7). At 2dai, 20 spots differed significantly between samples, while at 4dai this number decreased to 13 spots. ASM and Greenforce CuCa had a quite distinct protein profile at both time-points, being the ASM protein pattern more similar to that of the Control, particularly at 2dai.

**FIGURE 8:**
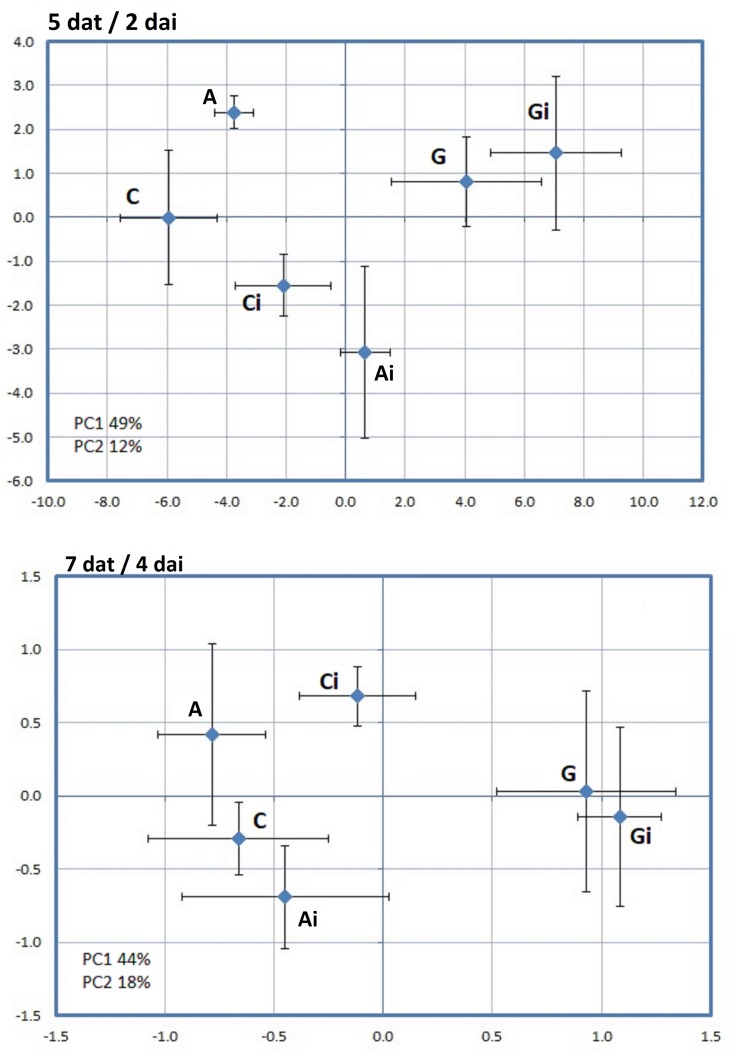
Principal component analysis (PCA) performed for the spots whose volume significantly changed in abundance (*p*-value < 0.05) between Greenforce CuCa (G), ASM (A), and Control (C) and treated and inoculation (i) with *H. vastatrix*, (Ci, Gi, Ai), at 5dat/2dai and 7dat/4dai.

**FIGURE 9:**
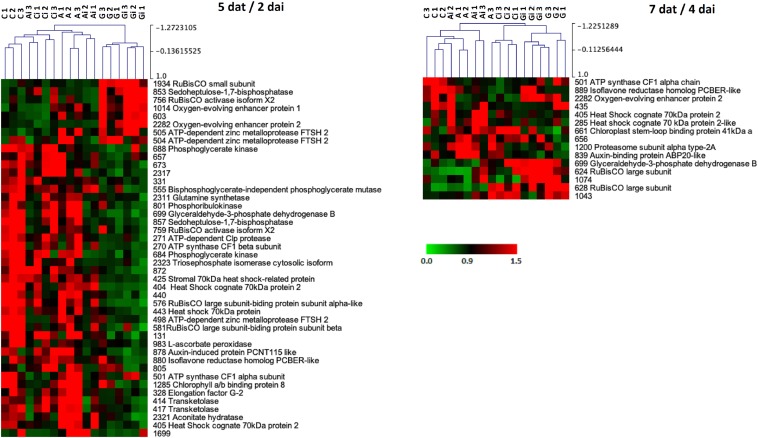
Hierarchical cluster analysis of the proteins that significantly changed in abundance (*p*-value < 0.05) between Greenforce CuCa (G), ASM (A), and Control (C) and treated and inoculation (i) with *H. vastatrix*, (Ci, Gi, Ai), at 5dat/2dai and 7dat/4dai. The signals are shown in a red-green color scale, from a gradient of red (higher expression) to green (lower expression).

A PCA analysis was further applied to the effects of all PRIs treatments (C x G x A x Ci x Gi x Ai) on the leaf proteome, aiming to reveal the factor that has the major influence on the dynamics of the leaf proteome, i.e., PRI treatments or *H. vastatrix* infection. Greenforce CuCa treatments (G and Gi) were clearly separated from Control and ASM (C, Ci, A, Ai) at 5dat/2dai and at 7dat/4dai (Figure 8). A hierarchical cluster analysis reinforced the PCA results and evidenced that: at 5dat/2dai 44 spots changed in abundance, while at 7dat/4dai this number decreased to 15 spots (Figure 9); the ASM treatment seems to induce fewer changes in the plant than the Greenforce CuCa treatments (at 5dat), exhibiting a profile more similar to Control. The protein profiles of the ASM treated and inoculated leaves (Ai) cluster together with those of inoculated Control leaves (Ci), while profiles of Greenforce CuCa treated and/or inoculated leaves cluster together (G and Gi).

### Proteins Differentially Expressed

The 58 proteins that changed in abundance due to the PRIs treatments and infection were mainly involved in: photosynthesis (27 spots, 47%), protein metabolism (8 spots, 14%), stress (7 spots, 12%), aerobic respiration (4 spots, 7%), and redox (4 spots, 7%) (Table 2). Considering the most discriminating time-point, 5dat/2dai (Figure 9), several proteins related to photosynthesis and to protein degradation positively contributed for the Greenforce CuCa cluster (G and Gi); oxygen-evolving enhancer proteins, OEE1 and OEE2 (#1014 and #2282 spots) and RuBisCO small subunit (#1934 spot) were only responsive to this treatment. Regarding the ASM treatment, other distinct proteins were induced, namely; RuBisCO chaperones (#576 and #581 spots), transketolase (#414 and #417 spots), phosphoglycerate kinase (#684 spots), HSP70 (#404 and #405 spots), acidic endochitinase-like protein (#905 spot) and one auxin-binding protein ABP20-like (#839 spot). Sedoheptulose-1,7-bisphosphatase (#853 and #857 spots) and ATP-dependent zinc metalloproteases (#498, #504 and #505 spots) changed with both PRIs treatments, but different putative isoforms were affected. Glyceraldehyde-3-phosphate dehydrogenase (GAPDH, spot #699) was also significantly increased by both treatments, but in a time-dependent way, earlier in ASM (5dat) than in Greenforce CuCa treatments (7dat).

### Physiological Analysis

**FIGURE 10:**
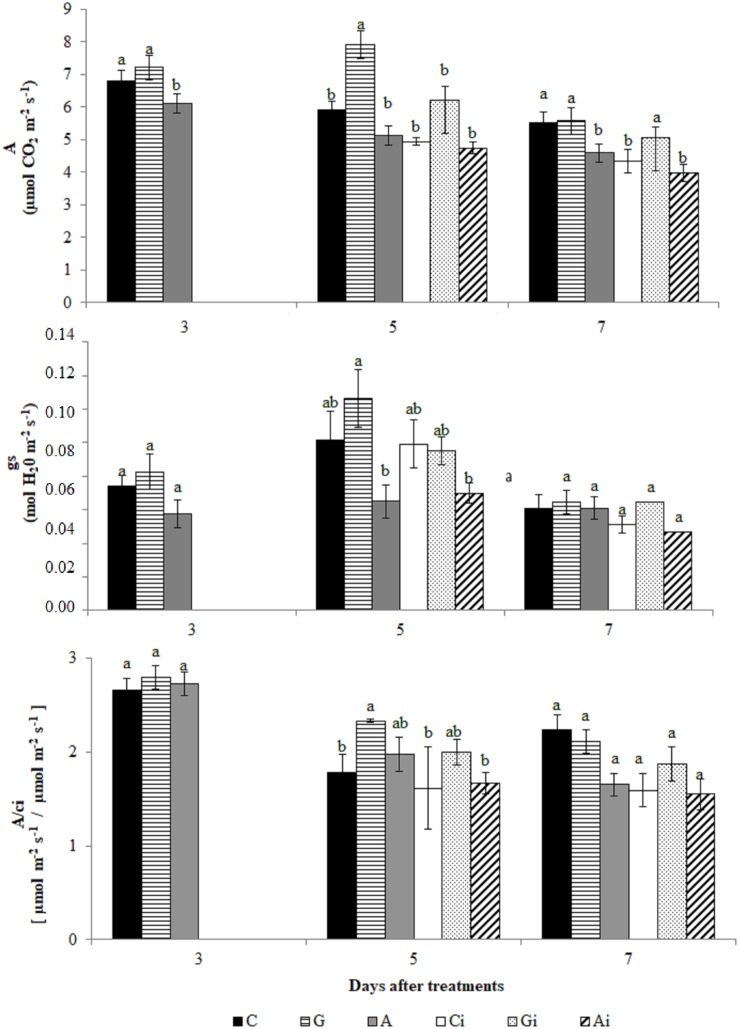
Evaluation of photosynthesis rate (*A*), stomatal conductance (*gs*) and *A/c_*i*_* in coffee leaves treated with the plant resistance inducers (PRIs) Greenforce CuCa (G), ASM (A), and control (C) and inoculated with *H. vastatrix* (i). Inoculation occurred 3 days after treatment with PRIs. The means from the treatments, at each evaluation time, followed by different letters were significantly different (*p* ≤ 0.05) according to Tukey test. Bars represent the standard error.

The physiological alterations observed in the coffee leaves due to the PRI treatments and *H. vastatrix* inoculation, were also studied. Greenforce CuCa treated leaves exhibited an increase in the photosynthetic rate (*A*) and stomatal conductance (*g*_*s*_) (at 5dat) when compared to ASM treatments (Figure 10). However, when comparing with Control, Greenforce CuCa showed a higher *A/c_*i*_* ratio. PRI treatments followed by *H. vastatrix* infection either decreased values or had no effect on the physiological parameters studied (Figure 10 and Supplementary Figure S2). Estimated values of water use efficiency (WUE, estimated as *A*/transpiration), mesophyll intercellular CO_2_ concentration (*c*_*i*_) and the ratio of intercellular to ambient CO_2_ concentrations (*c_*i*__/_c_*a*_*) did not change significantly between treatments/inoculation (Supplementary Figure S2).

### Biochemical Analyses

**FIGURE 11:**
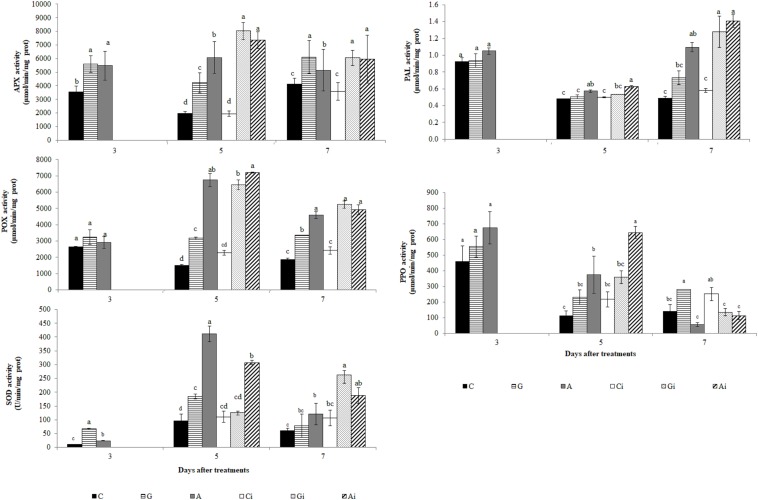
Evaluation of APX, POX, SOD, PAL and PPO activities in coffee leaves treated with the PRIs Greenforce CuCa (G), ASM (A), and control (C) and inoculated with *H. vastatrix* (i). Inoculation occurred 3 days after treatment with PRIs. The means from the treatments, at each evaluation time, followed by different letters were significantly different (*p* ≤ 0.05) according to Tukey test. Bars represent the standard error.

At the biochemical level the PRI treatments increased the activity of all the enzymes studied (APX, POX, SOD, PPO, and PAL) when comparing to a mock-treated control (Figure 11). An increase in H_2_O_2_ accumulation was only observed with the Greenforce CuCa treatment, at 5dat/2dai (Supplementary Figure S3). Upon *H. vastatrix* infection of PRI treated leaves the same pattern of response for all enzymes was observed (relatively to infected control, Ci) except for PPO, whose activity decreased. However, no significant differences were observed between the infected and non-infected controls (Ci and C). The highest values for the PAL activity were obtained at 7dat/4dai, contrary to what was observed for all the other enzymes, whose activity increase at 5dat/2dai.

## Discussion

### Coffee Leaf Proteome

When analyzing the whole coffee leaf proteome the proteins mostly represented were annotated as related to energy production and carbon fixation (“photosynthesis”) followed by “protein metabolism” (essentially protein synthesis) and “stress” response, similarly to what was found in the leaf proteomes of several other plants (Supplementary Table S5). These results contrast with those of the coffee leaf apoplast proteome previously studied (Guerra-Guimarães et al., 2014, 2015) which evidence protein degradation as the main biological process. It is notorious that among the proteins identified in the whole coffee leaf proteome, circa 9% were annotated as extracellular proteins, namely chitinase-like and cupin-like proteins that had already been referred for the coffee leaf apoplast proteome (Guerra-Guimarães et al., 2014, 2015).

### Effects of the PRI Treatments and *H. vastatrix* Infection

As far as we know, this is the first report on the proteomic analysis of the effect of PRIs on the leaves of a woody plant (coffee) before and after infection with a biotrophic pathogen (*H. vastatrix*). The PRIs ASM and Greenforce CuCa had been shown to reduce the incidence of CLR in the fields (Costa et al., 2014; Silva et al., 2019). We now confirmed their protective effect under controlled conditions, Greenforce CuCa showing a higher protective effect than ASM (Supplementary Table S1A). When analyzing the molecular mechanisms involved in such induced resistance, we verified that both PRIs modulate the primary metabolism (photosynthesis) and protein degradation, although through different proteins (Figure 9 and Graphical Abstract). This is in line with results of other authors, either in coffee (De Nardi et al., 2006) or in other plants (Barilli et al., 2012; Arasimowicz-Jelonek et al., 2013; Anup et al., 2015; Balmer et al., 2015). PRIs cause minimal changes in primary metabolism switching the plant defense mechanism to a standby state, which according to these authors is important in the defense against pathogens. We observed that at early stages of the *H. vastatrix* infection fewer alterations were induced in the leaf proteome of Greenforce CuCa treated leaves than by ASM. On the other hand, Greenforce CuCa also showed a better physiological performance than ASM, as expressed by an increase of about 20% in stomatal conductance (*g*_*s*_) and in the photosynthetic rate (*A*). These results might suggest that the differences in the carbon assimilation metabolism may have implications in the defense mechanisms induced by the two PRIs against *H. vastatrix*.

Our proteomic data did not show major changes in proteins of redox and secondary metabolism, but we did observed an increase in the activity of several related enzymes namely, APX, POX, SOD, PPO, and PAL. The integration of proteomic and biochemical data, point out for an additional level of regulation since different activities could be related with post-translation regulation of proteins and not with changes in protein abundance (Yin et al., 2019). Furthermore, 2-DE capacity to distinguish small changes of low abundant proteins is limited, which make harder the correlation of both data sets. The increases in activity of APX, POX, SOD, PPO, and PAL due to the PRIs treatments and to infection can be related with the defense responses. It is known that the redox enzymes (e.g., APX, POX, and SOD) have a role in eliminating excessive ROS caused by pathogen infection, while the increases in PPO and PAL activities might point out for modifications in secondary metabolism, eventually leading to lignin formation. In fact, increase in POX, SOD and PAL activities, accumulation of phenolics and cell wall modification/lignification at the infection sites were previously observed during the coffee resistance to *H. vastatrix* (Silva et al., 2002, 2008; De Nardi et al., 2006; Guerra-Guimarães et al., 2009, 2015; Leitão et al., 2011).

### Relevance of Proteins Differentially Affected by PRI Treatments

It is notorious the lack of similarity in the protein profiles induced in the coffee leaves by Greenforce CuCa and ASM, what suggests the modulation of different metabolic pathways by these PRIs. The exceptions were glyceraldehyde-3-phosphate dehydrogenase (GAPDH) and sedoheptulose-1,7-bisphosphatase (SBPase). GAPDH respond to both PRIs in a time-dependent way, while putative SBPase isoforms were distinctly induced.

The annotation of GAPDH indicated its localization in the chloroplast, with a probable participation in the reductive pentose phosphate pathway (Calvin-Benson cycle). It is known that GAPDH is a component of the triose phosphate/pentose phosphate pool, important in several biosynthetic pathways that require carbon skeletons (Lim et al., 2013). It has also been considered that this enzyme has a role in defense responses, namely, as a regulator of ROS accumulation and cell death in Arabidopsis infected with *Pseudomonas syringae* (Henry et al., 2015). GAPDH respond positively to pathogen infection and to PRIs treatments (McGregor et al., 2009; Mutuku and Nose, 2012; Lemaître-Guillier et al., 2017), indicating a possible involvement of this enzyme in the resistance induction in coffee. SBPase has been classified as a key enzyme in photosynthetic carbon fixation and growth (Lefebvre et al., 2005; Nilo-Poyanco et al., 2013). It was shown to be positively related with resistance of peanuts to *Phaeoisariopsis personata* (Kumar and Kirti, 2015) and to be decreased in susceptible mulberry when infected with mulberry dwarf phytoplasma (Ji et al., 2009). SBPase seems to be also important during coffee resistance induction, but further studies are needed in order to reveal the role of the distinct putative isoforms detected.

One group of the proteins specifically affected by the ASM treatment were the stress-related proteins HSP70 probably involved in housekeeping folding metabolism. Indeed, it was described in leaves of *V. vinifera* the increase in abundance of HSPs proteins due to a PRI treatment (Lemaître-Guillier et al., 2017). HSP70 proteins are ATP-dependent chaperones considered to maintain protein homeostasis by their involvement in: the proper folding of nascent synthesized proteins, the prevention of protein aggregation, the translocation of proteins across membranes, and the protein targeting to degradation (Sarkar et al., 2013). As a consequence of their important role in protein homeostasis they also participate in plant immunity through the quality control of the pattern recognition receptors (PRRs) (Park and Seo, 2015). These PRRs are essential in the detection of pathogen molecules (PAMPs-pathogen associated molecular patterns), as for instance, in the resistance of *Nicotiana tabacum* against *Ralstonia solanacearum* (Maimbo et al., 2007). Chitinase-like protein and an auxin-binding protein ABP20-like (germin-like protein) were also induced by ASM. Chitinases (PR-3, 4, 8, 11) are well-characterized glycosyl hydrolases (GH18) with potential to degrade PAMPs, limiting pathogen growth and functioning as signals for resistance responses (Silva et al., 2006; Guerra-Guimarães et al., 2009, 2015). The germin-like protein family is a considerably heterogeneous group exhibiting three different enzymatic activities, oxalate oxidase, ADP-glucose pyrophosphatase or phosphodiesterase and superoxide dismutase (SOD) (Dunwell et al., 2008). The induction of the germin-like proteins may contribute to the significantly higher SOD activity detected due to the ASM treatment. The germin-like proteins can have a role in the oxidative crosslinking of cell wall proteins around the site of infection (Bradley et al., 1992; Silva et al., 2008). Crosslink between phenolic compounds, plant cell wall polysaccharides and proteins enhance the protection of the cell wall to digestion by microbial degrading enzymes and, thus, increase the global resistance to fungi (Bily et al., 2003). The apoplastic localization of these proteins, in combination with the H_2_O_2_ generating SOD activity, offers a role in cell-wall fortification (Rietz et al., 2012). Indeed, the modulation of apoplastic chitinases, germin-like proteins and SOD during the early stages of *C. arabica*-*H. vastatrix* interactions have been reported (Guerra-Guimarães et al., 2015).

Greenforce CuCa treatment consistently induces changes in oxygen-evolving enhancer proteins (OEE1 and OEE2), RuBisCO activase and RuBisCO small subunit, which might improve the coffee response to rust. Potato treated with different PRIs, also shown relevant variations in OEE2 protein abundance (Arasimowicz-Jelonek et al., 2013). It was proposed that OEE1 and OEE2 may have a role in defense (Heide et al., 2004; Coppola et al., 2013). In Arabidopsis OEE1 and OEE2 exhibited properties of thioredoxins, which are positive regulators of plant-induced defense responses (Tada et al., 2008). The involvement of OEEs in ROS detoxification as a response to biotic stress (Heide et al., 2004; Coppola et al., 2013) is in line with our findings. These results, together with the increased activity of APX, POX, SOD and H_2_O_2_ production, strongly suggests a role of the Greenforce CuCa treatment in the redox homeostasis during the *H. vastatrix* infection.

The RuBisCO activase and the RuBisCO small subunit, regulators of RuBisCO activity and stability (Buchanan et al., 2015), were described as involved in the resistance of grapevine to *Plasmopora viticola* (Lemaître-Guillier et al., 2017). Differences in both RuBisCO activase and RuBisCO small subunit had been used to distinguish between two genetically close inbred tomato lines with opposite responses to TYLCV virus infection (one resistant and one susceptible) (Moshe et al., 2012). Moshe et al. (2012) further highlighted the decrease in abundance of ATP-dependent zinc metalloproteases (FtsH, Filamentation Temperature-Sensitive protein H) in susceptible inbred tomato lines. The effect of Greenforce CuCa on protein degradation was evidenced by the increase in abundance of FtsH, proteases known to be involved in different biological processes namely: thylakoid membrane organization, proteolysis and ROS metabolism (Kato et al., 2009; Tibiletti et al., 2016). In addition, it was also suggested that these proteins might act as chaperones (Adam and Clarke, 2002). Greenforce CuCa treatment is outstanding in inducing the increase in abundance of the above referred proteins what, together with an increase in the photosynthetic rate, is important for the coffee plant to deal with CLR.

## Conclusion

We used a proteomic approach in order to shed light on the effect of the PRIs, Greenforce CuCa and ASM, on the coffee leaf metabolism. The aim was to improve knowledge on the resistance mechanisms induced by the PRIs against CLR. Proteomic adjustments mainly related to photosynthesis, protein metabolism and stress responses were shown. However, the proteins affected by Greenforce CuCa were different from those affected by ASM. It was further observed that Greenforce CuCa reinforces the redox homeostasis of the leaf, while ASM seems to affect preferentially the secondary metabolism and the stress-related proteins. A link between the both PRI treatments, the primary metabolism and the defense responses was also evidenced. While both PRIs prepare the plant to resist CLR, they induce distinct defense mechanisms. The identification of components of the plant primary metabolism (essential for plant growth and development) that, simultaneously, participate in the plant defense responses (e.g., SAR) could open new perspectives for a plant breeding program.

## Data Availability Statement

The mass spectrometry proteomics data have been deposited to the ProteomeXchange Consortium via the PRIDE partner repository with the dataset identifier PXD016012.

## Author Contributions

MR and LG-G conceived and designed the research and were involved in funding acquisition. KP and JS conducted all experiments, and collected the biological material. KP and RT conducted the 2-DE electrophoresis and image analysis. SP and JR carried out the MS-based spot identification. CP, IC, and LG-G conducted the statistical analyses and functional annotation of the identified proteins. JS and MC extracted and analyzed all the physiological parameters. JS and AM extracted and analyzed the data from the biochemical assays. CP, CR, and LG-G wrote the manuscript. All authors have read and approved the final version of the manuscript.

## Conflict of Interest

The formulation “Greenforce CuCa” is patent pending. There is no commercial interest regarding this patent since the purpose of INCT coffee (the intellectual property owner) is to make it freely available to coffee growers. The authors declare that the research was conducted in the absence of any commercial or financial relationships that could be construed as a potential conflict of interest.

## Footnotes

1 www.matrixscience.com

2 http://www.ncbi.nlm.nih.gov

3 http://mapman.gabipd.org/web/guest/mapman

4 http://mapman.gabipd.org/web/guest/app/mercator

5 http://www.geneontology.org

6 http://www.blast2go.com

7 https://rostlab.org/services/loctree2/
