# Supplementary figures and images for: Primary Metabolism Is Distinctly Modulated by Plant Resistance Inducers in Coffea arabica Leaves Infected by Hemileia vastatrix
Source: Front Plant Sci. 2020 Mar 20;11:309. doi: 10.3389/fpls.2020.00309 (PMC7099052; doi:10.3389/fpls.2020.00309)

Control, 3 days

**B**

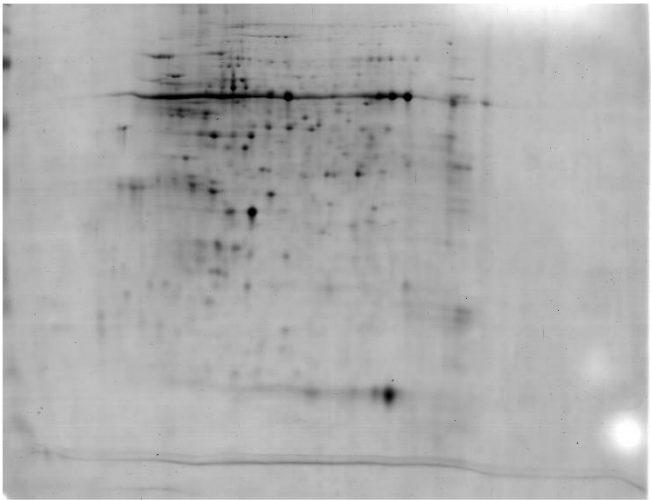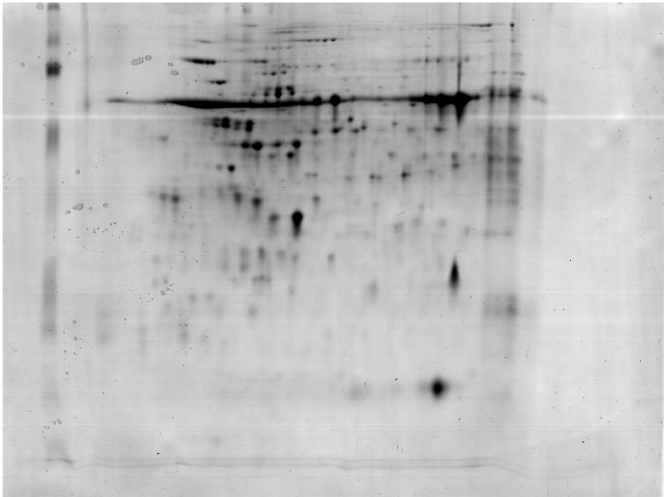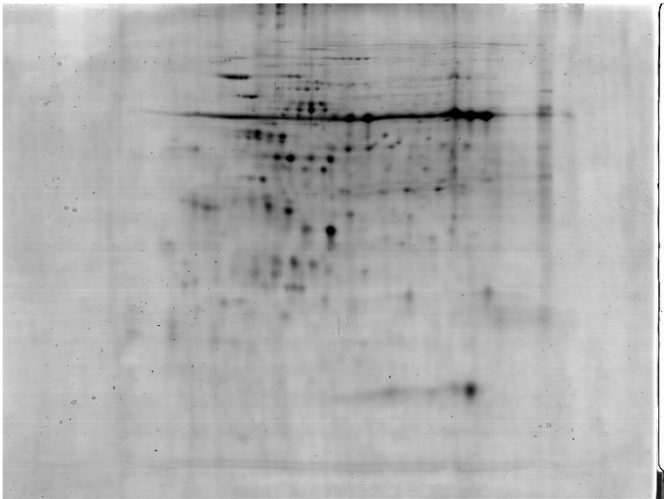

**B**

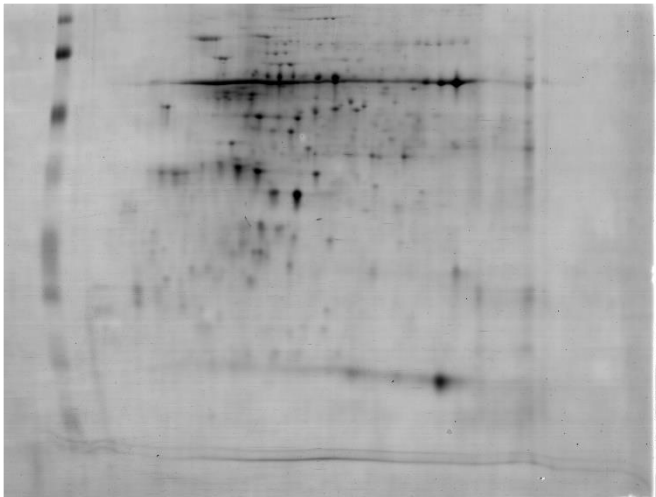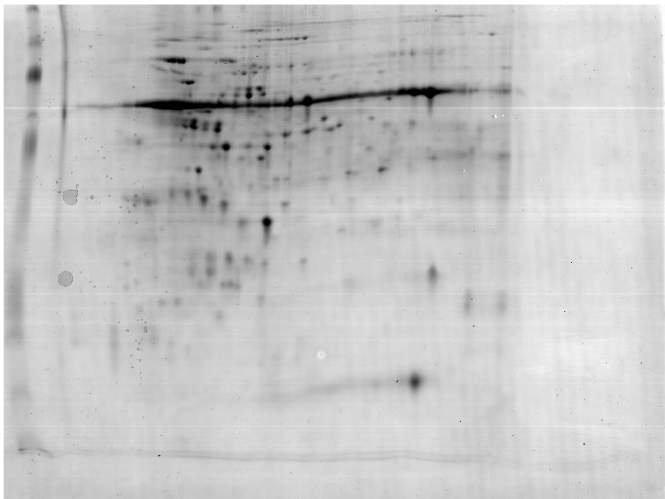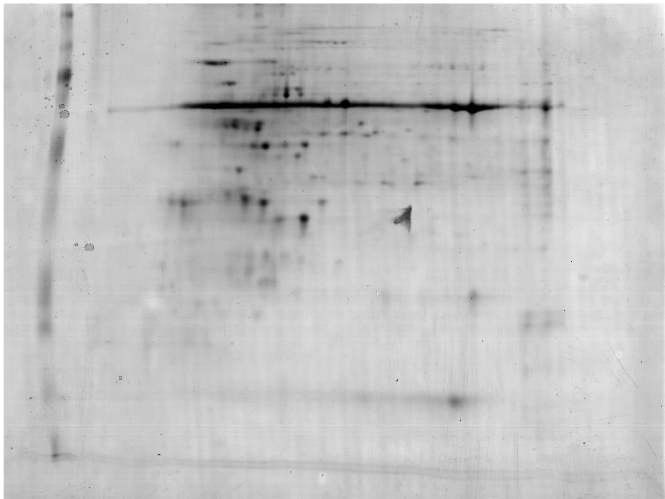

**B**

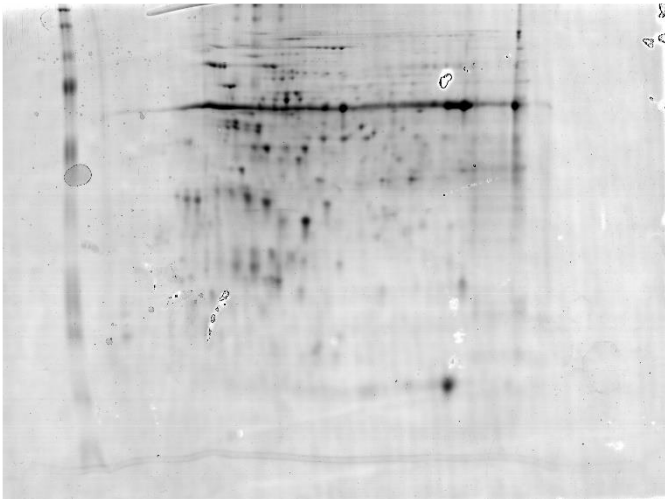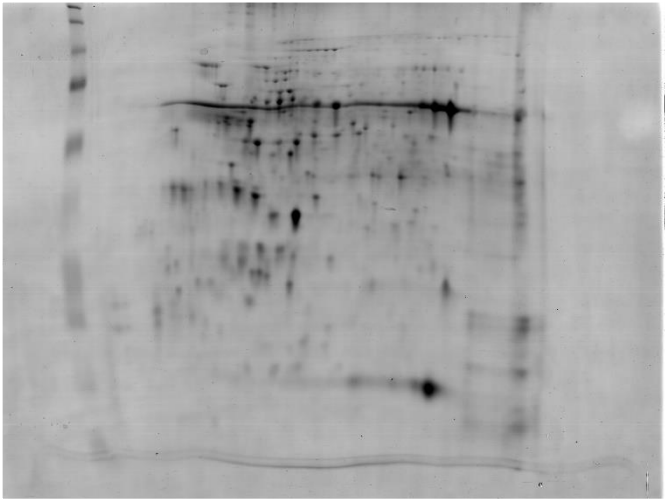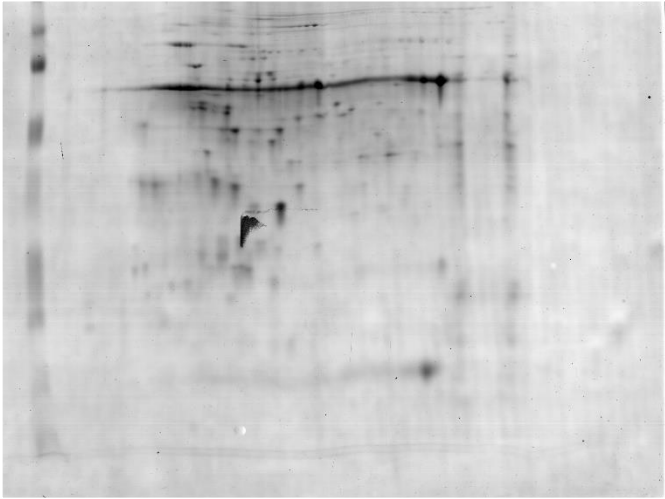

Control, 5 days

**B**

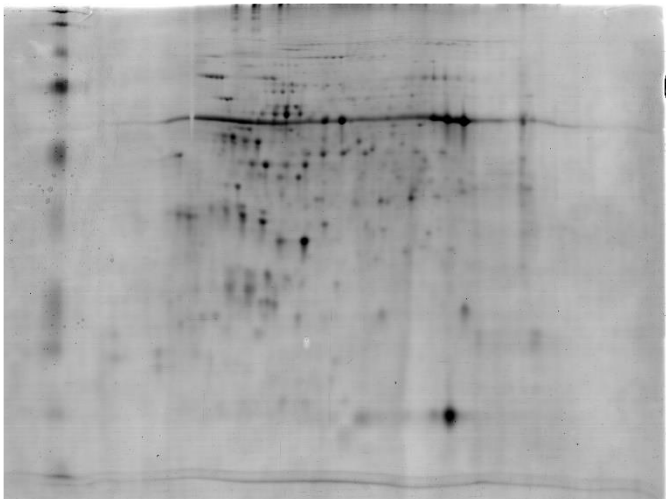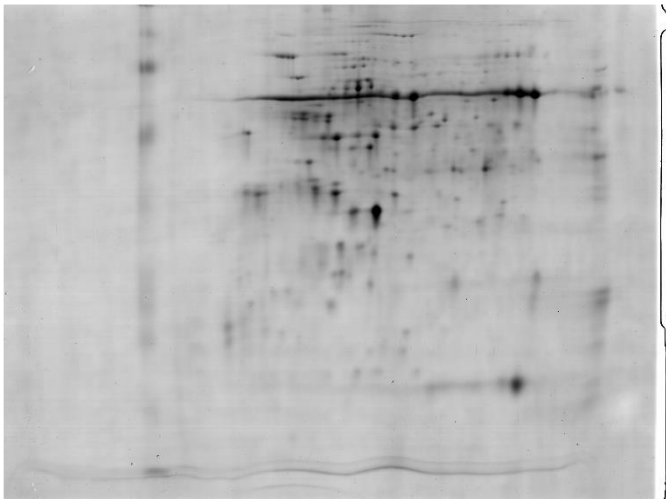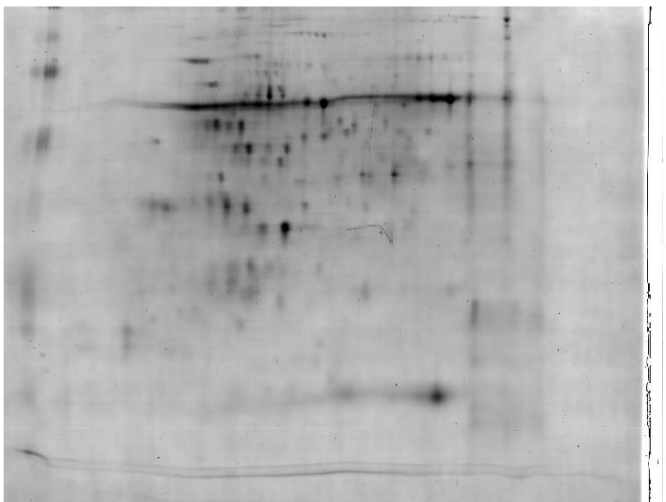

**B**

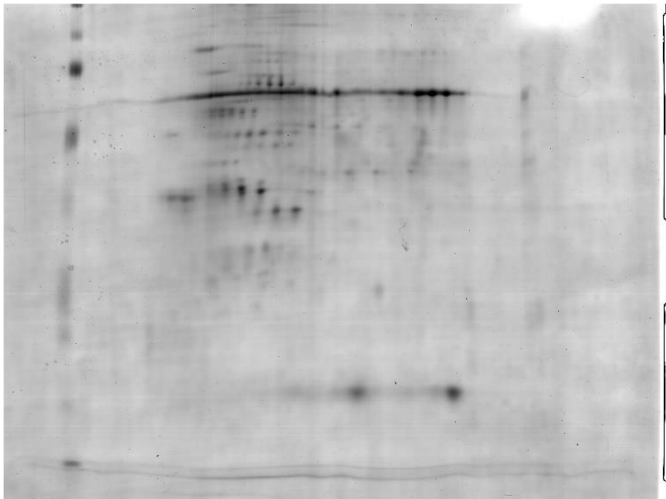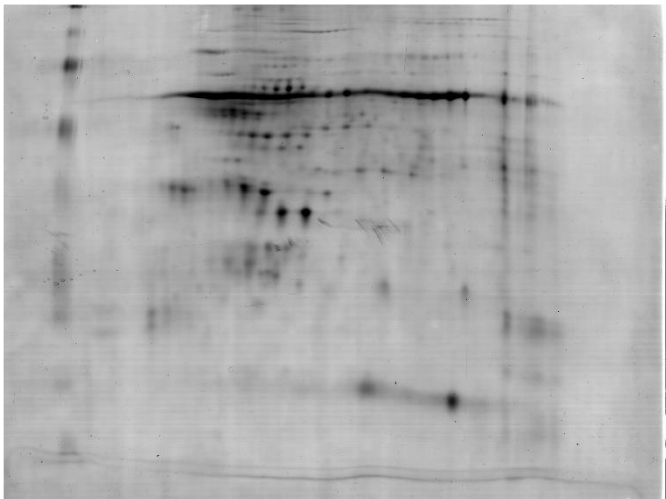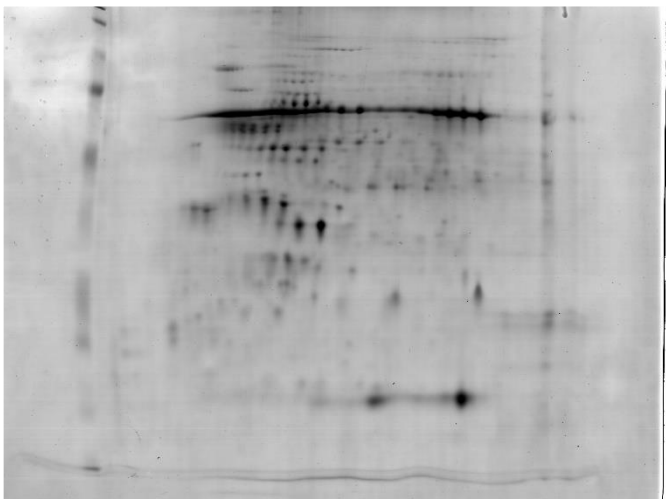

**B**

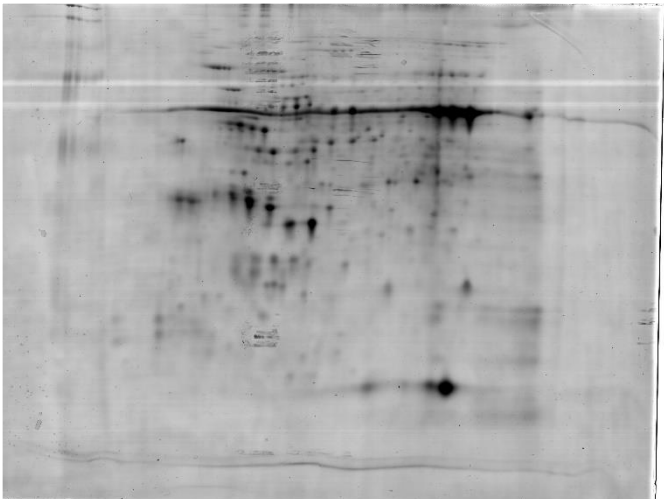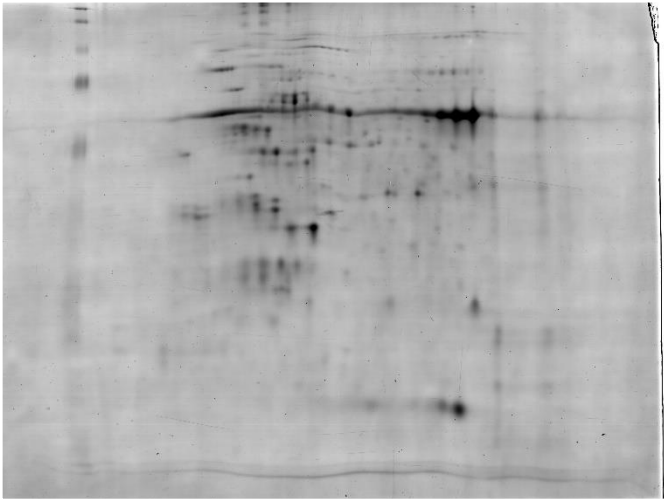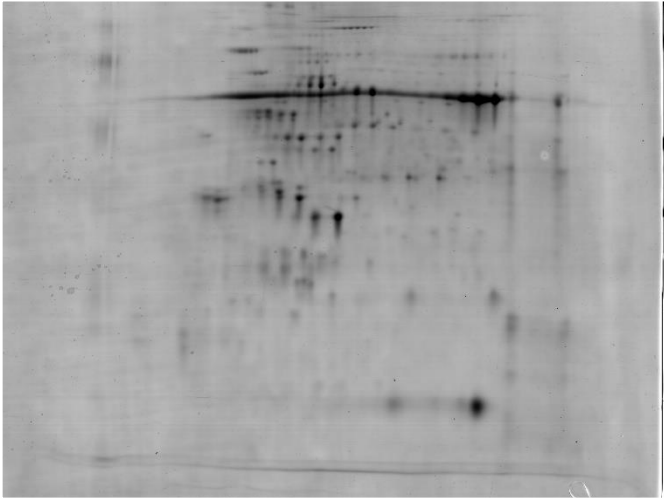

Inoculated Control, 5 days

**B**

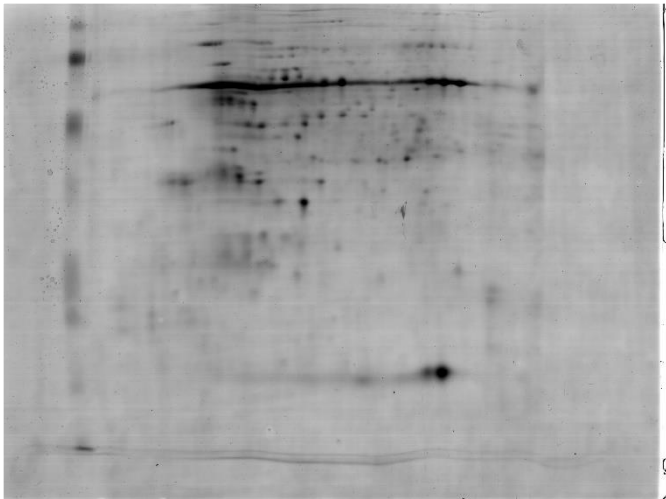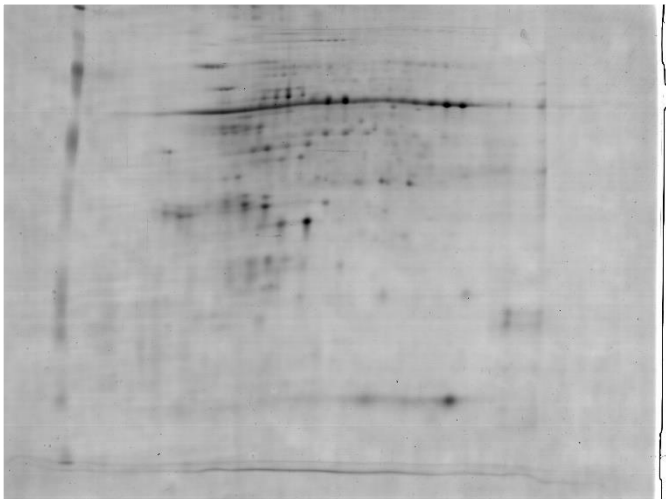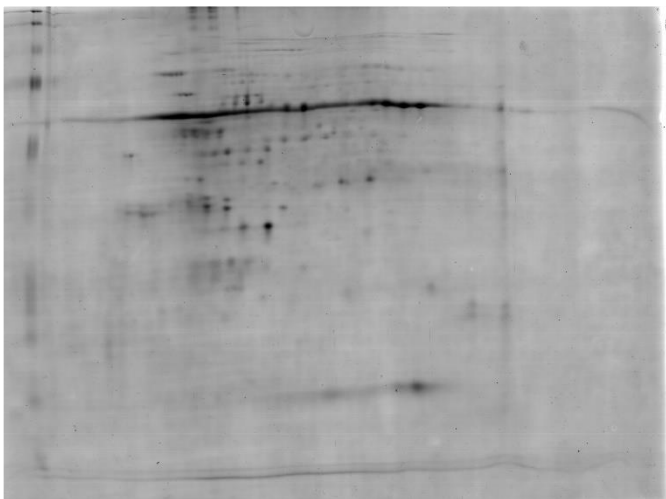

**B**

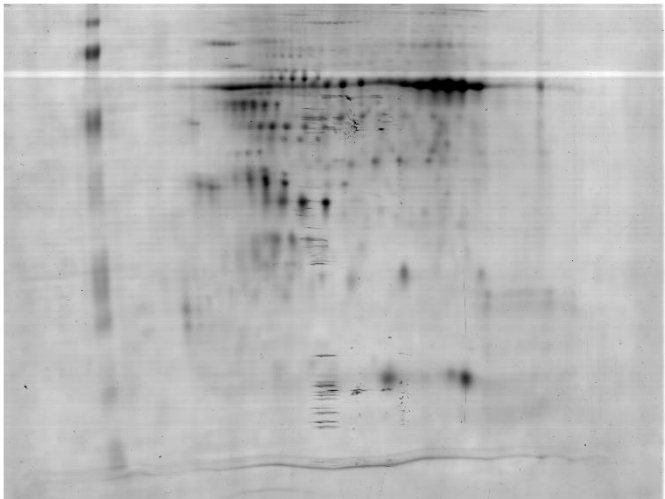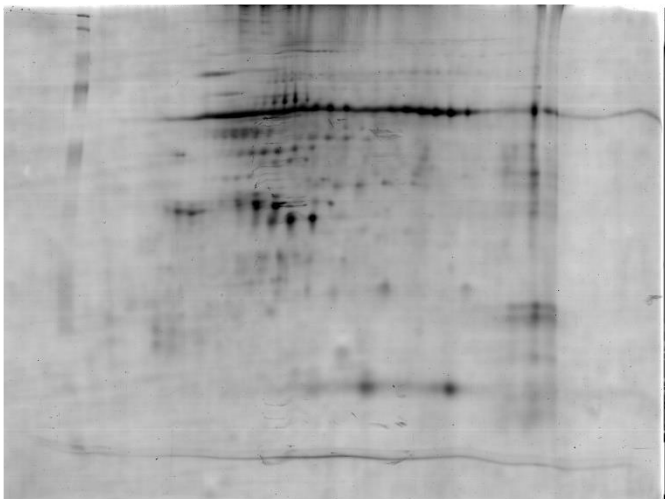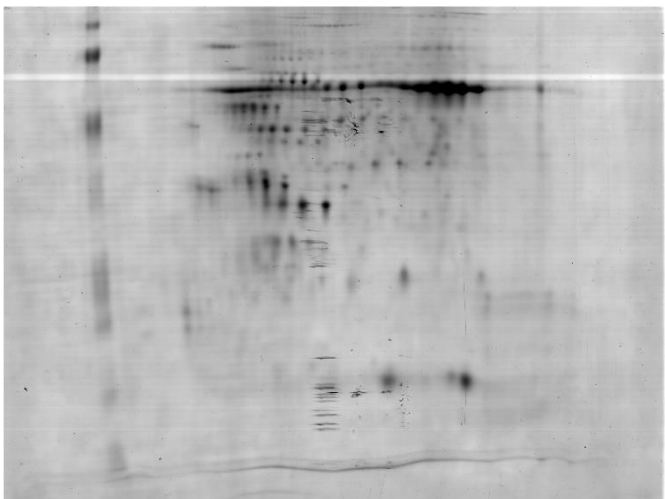

**B**

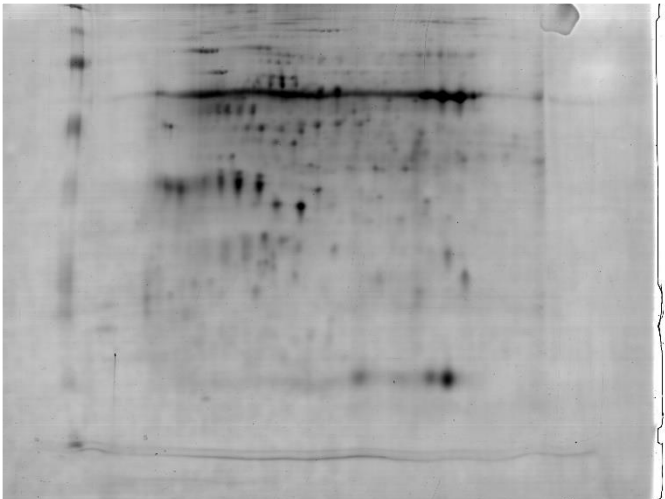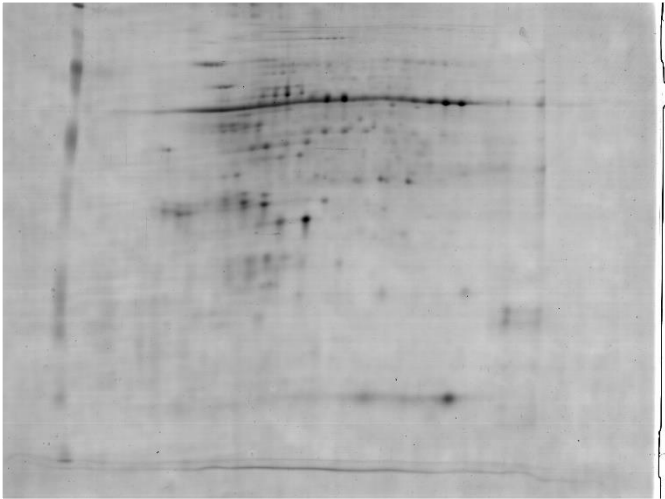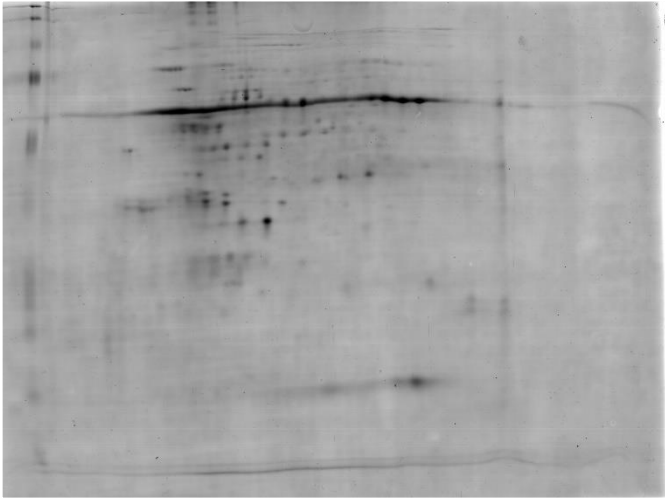

Control, 7 days

**B**

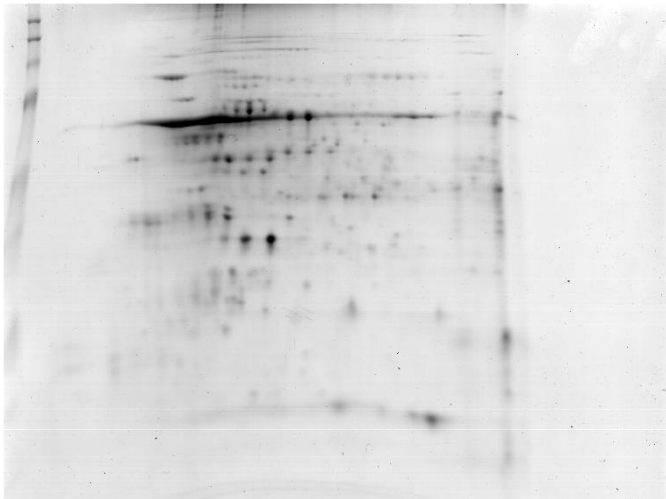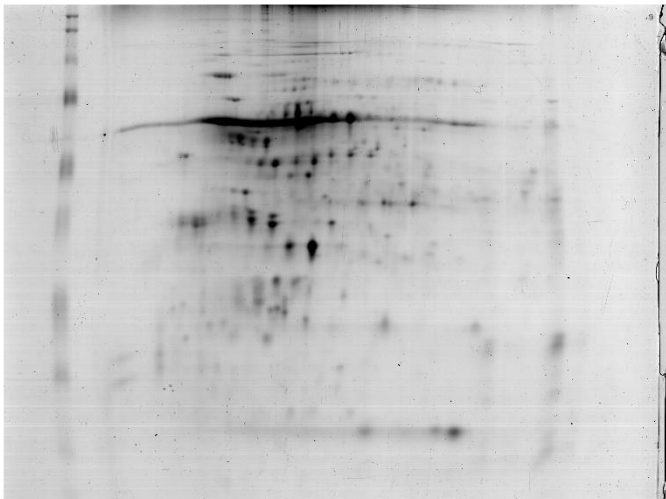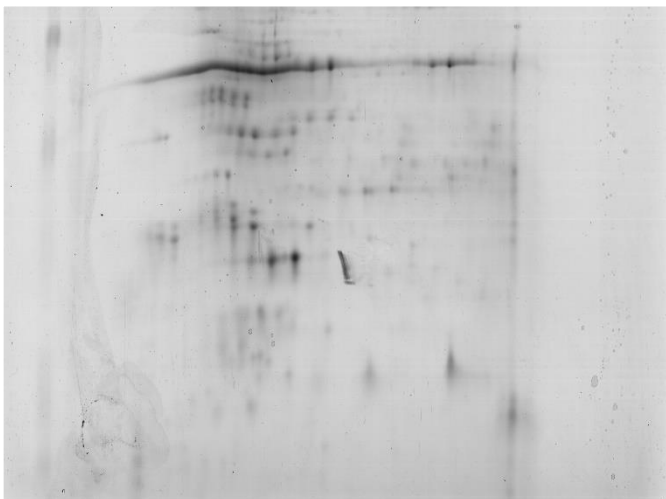

**B**

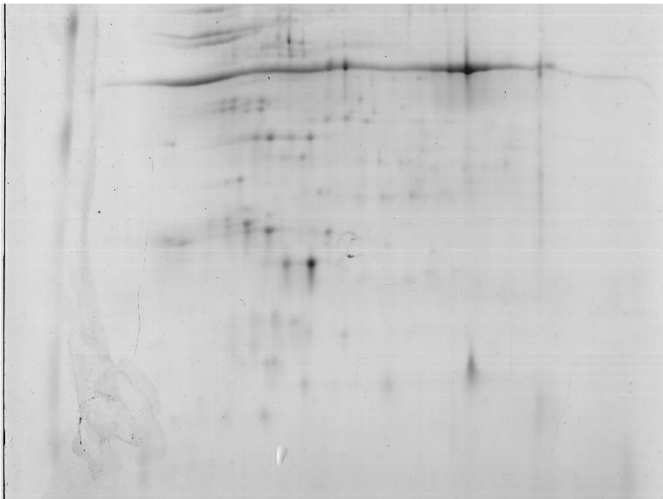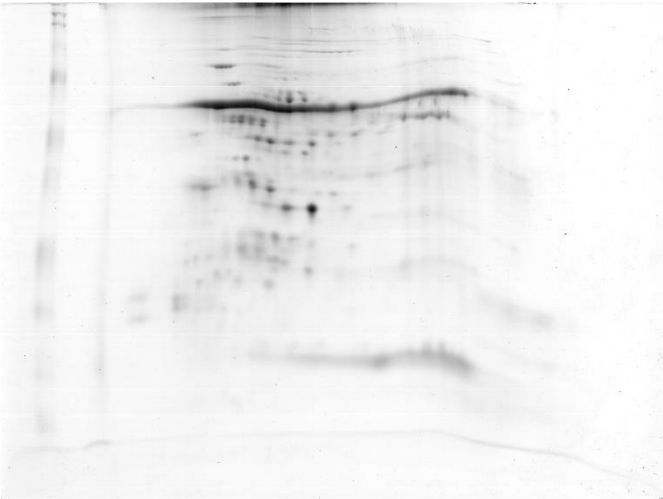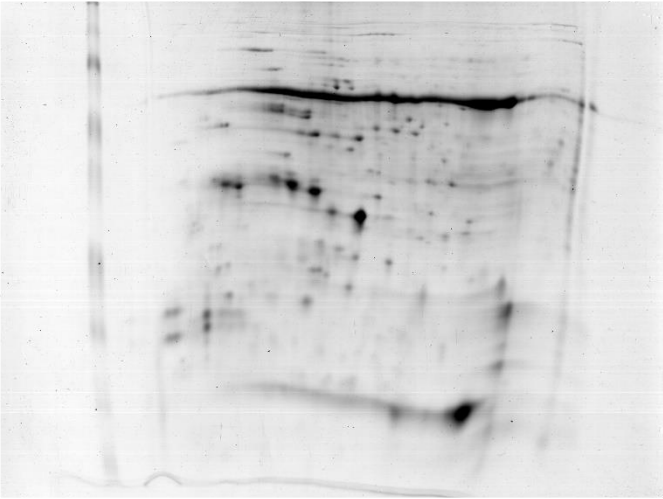

**B**

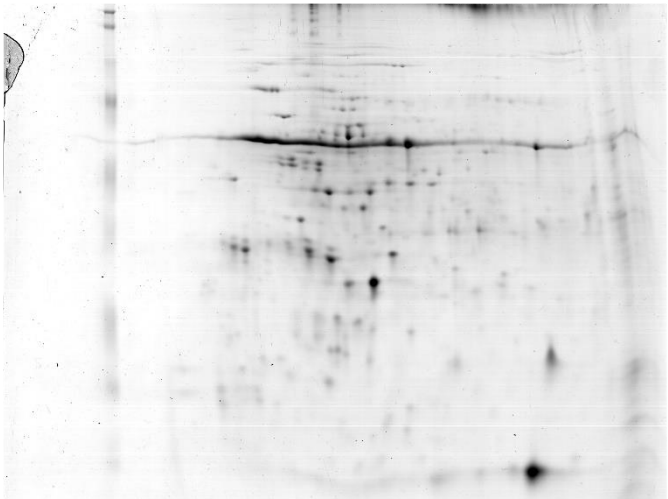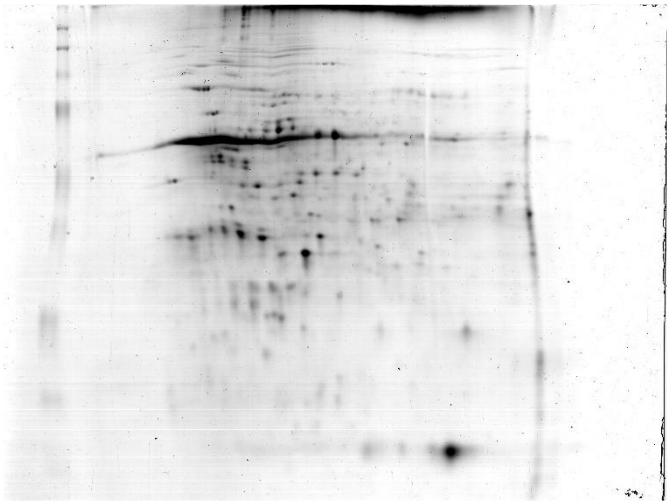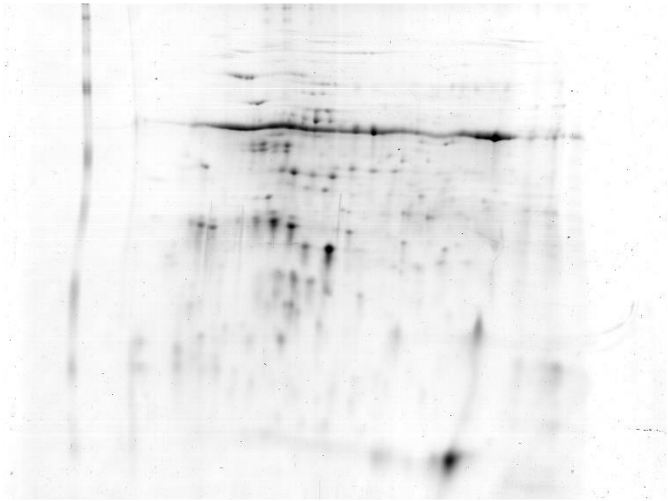

**B**

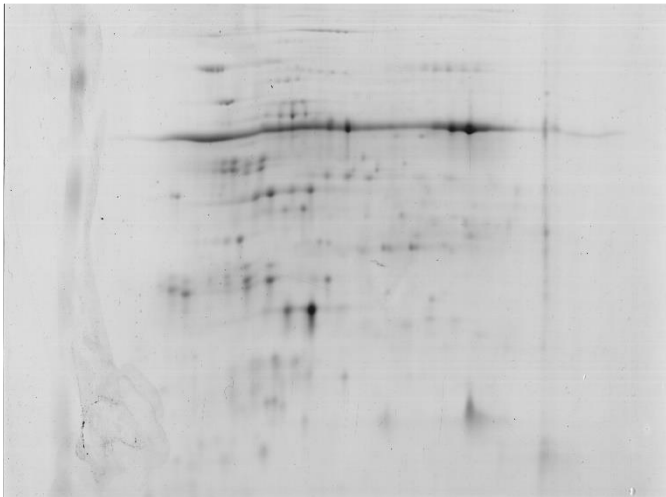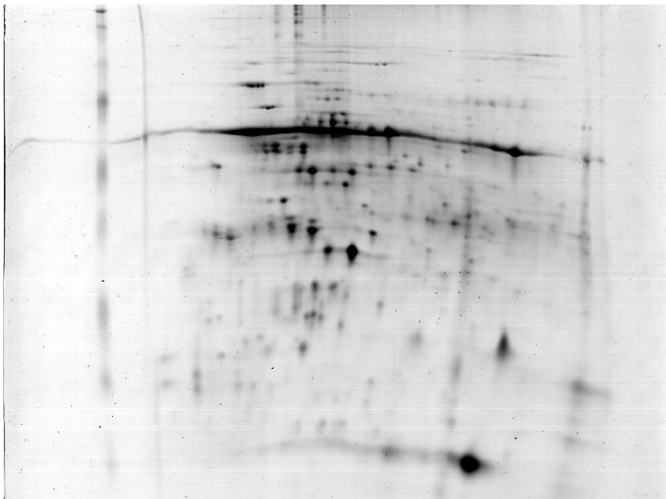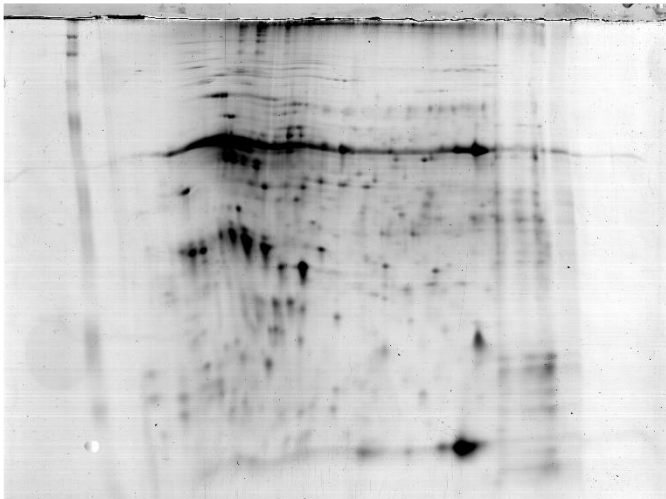

**B**

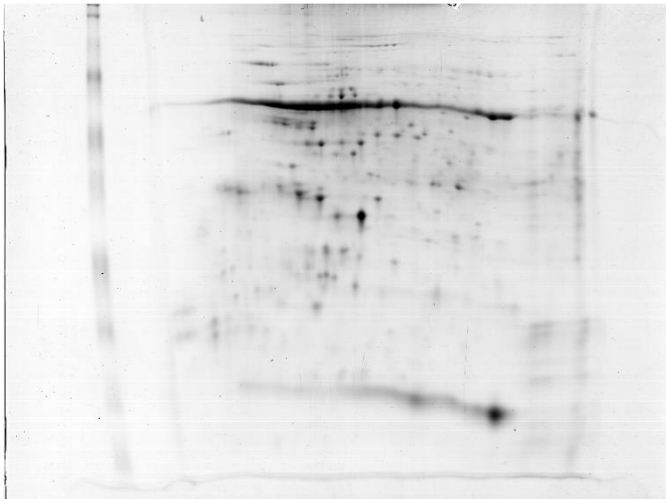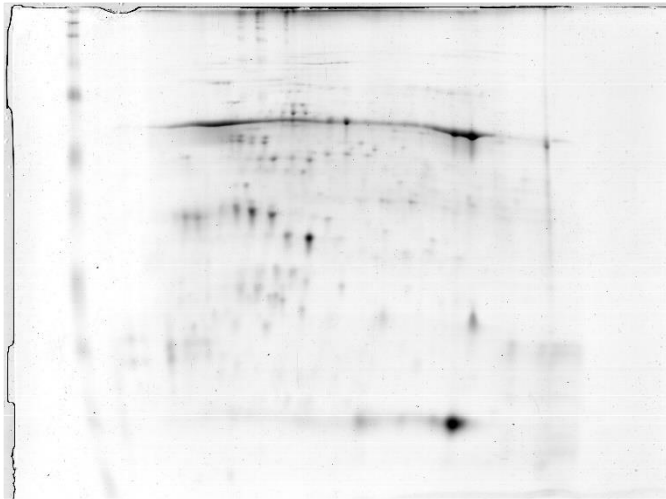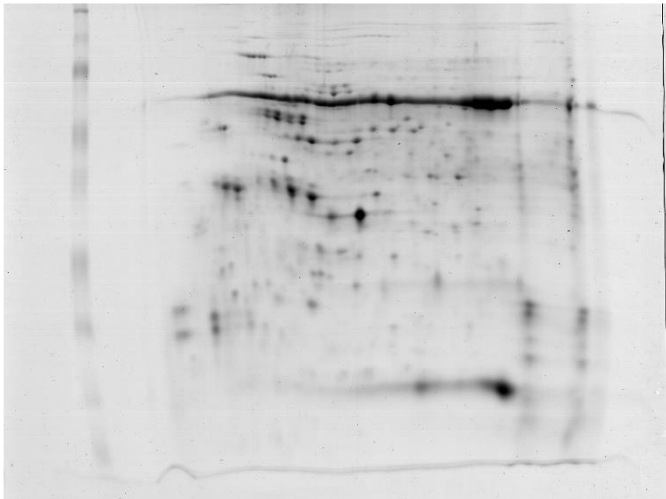

**B**

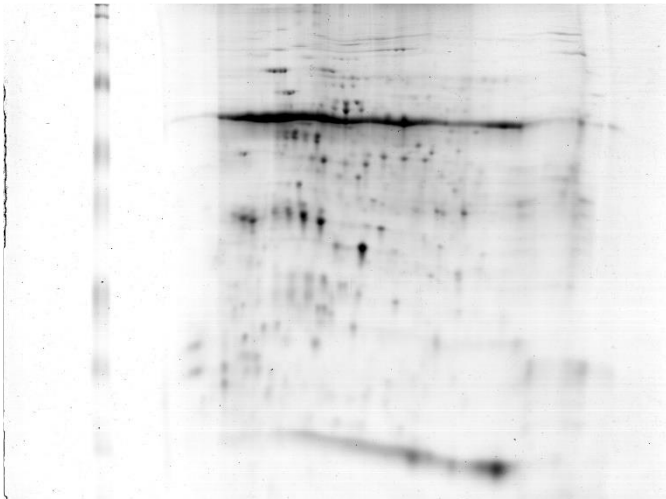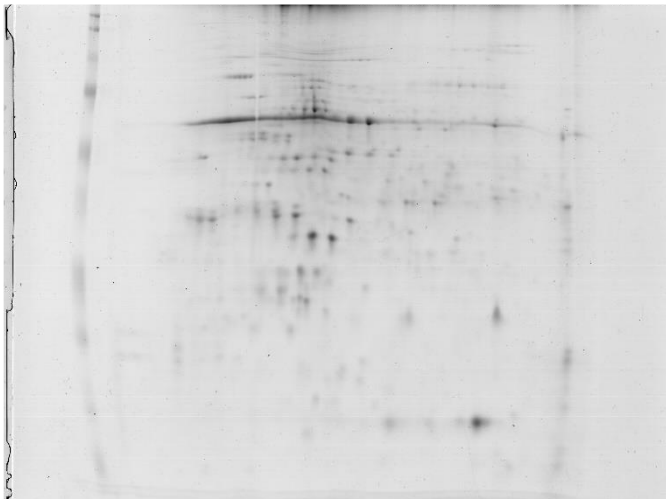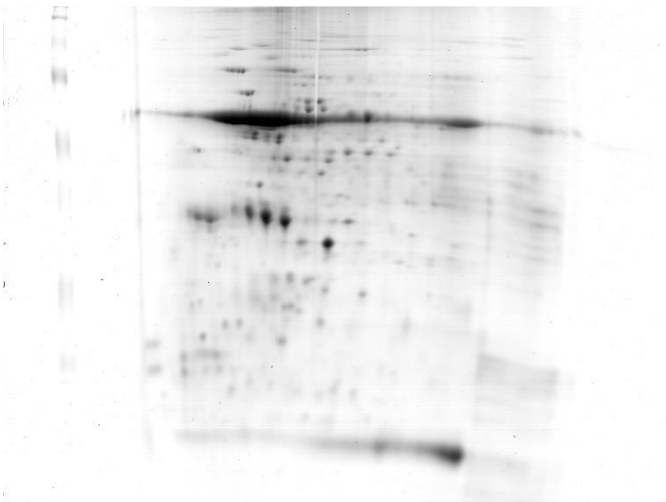

Supplement: Supplementary file 1 [file Image_1.pdf]

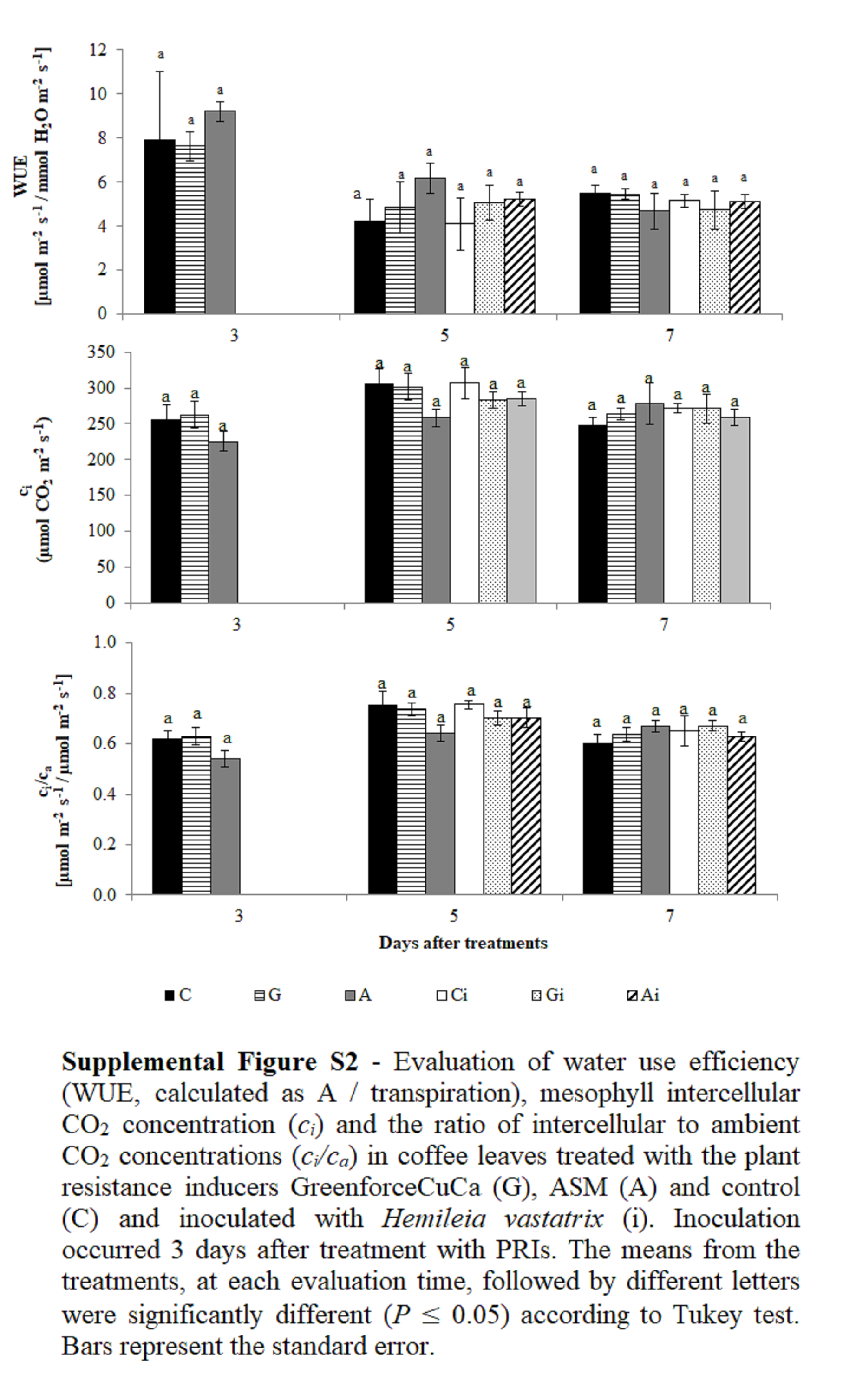

Supplement: Supplementary file 2 [file Image_2.TIF]

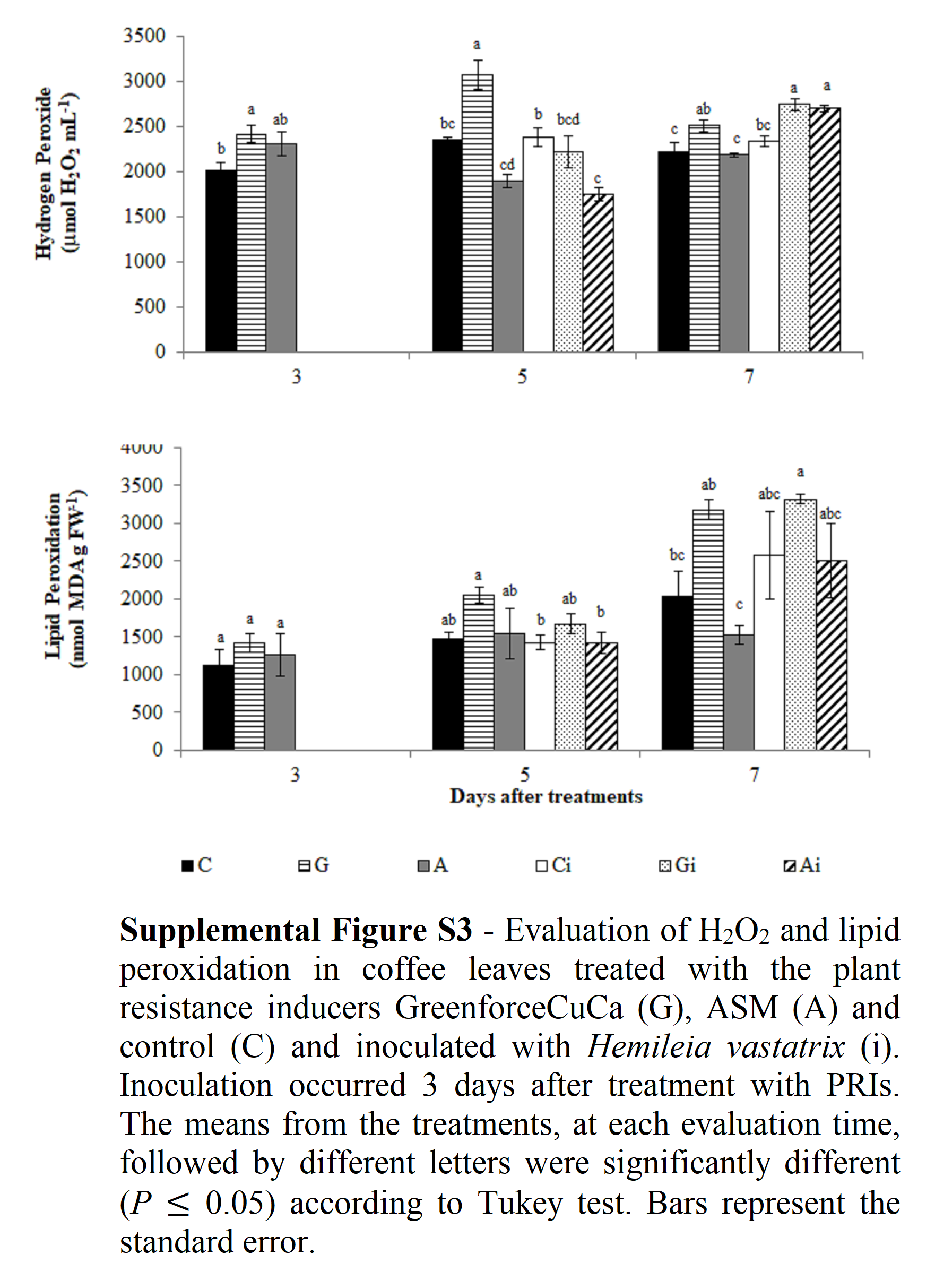

Supplement: Supplementary file 3 [file Image_3.TIF]
