# Supplementary material for: Primary Metabolism Is Distinctly Modulated by Plant Resistance Inducers in Coffea arabica Leaves Infected by Hemileia vastatrix
Source: Front Plant Sci. 2020 Mar 20;11:309. doi: 10.3389/fpls.2020.00309 (PMC7099052; doi:10.3389/fpls.2020.00309)
Supplement: Supplementary file 4 [file Table_1.pdf]

Supplemental Table S1A. Effect of treatments on the area under the disease severity progress curve (AUDsPC) on the coffee rust disease

**Treatments**	**AUDsPC**[*]	**Disease control**[T]
Control (mock-treated)	92.0 b	-
Greenforce CuCa	54.3 a	41%
ASM	70.8 ab	23%

[*]The rust disease severity assessment was done by visual evaluation of the inoculated leaves after the appearance of the first symptoms (around 30 days after inoculation) and every 7 days, in a total of five evaluations, according to a diagrammatic scale (Capucho et al. 2011). The observed indexes of severity were transformed in the area under the corresponding disease severity progress curve (AUDsPC) as proposed by Shaner and Finney (1977). Any two means followed by the same letter in the column were not significantly different at the 0.05 probability level according to the Tukey test.

[T]Disease control (%) means the amount of the disease which was controlled by treatments when compared to the mock-treated control.

Supplemental Table S1B. Effect of treatments on the CLR disease severity

	**CLR Disease Severity Index**				
	**Number of Evaluations**				
**Treatments**	**1**	**2**	**3**	**4**	**5**
Control	3.42 ±0.83 b	3.25 ±0.80 b	2.88 ±0.83 a	3.09 ±0.70 a	4.38 ±0.92 a
Greenforce CuCa	1.33 ±0.19 a	1.27 ±0.14 a	2.19 ±0.49 a	2.25 ±0.38 a	2.75 ±0.42 a
ASM	2.66 ±0.32 ab	2.38 ±0.38 ab	2.33±0.43 a	2.75 ±0.66 a	2.62 ±0.52 a

Rust disease severity index was assessed by visual evaluation of the inoculated leaves after the appearance of the first symptoms (around 30 days after inoculation) and every 7 days, in a total of five evaluations, according to a diagrammatic scale (Capucho et al. 2011). The means from the treatments ± standard error, at each evaluation, followed by different letters were significantly different $P \leq 0.05$ according to Tukey test.

Capucho, A. S., Zambolim, L., Duarte, H. S. and Vaz, G. R. (2011), Development and validation of a standard area diagram set to estimate severity of leaf rust in *Coffea arabica* and *C. canephora*. *Plant Pathology* 60, 1144-1150. doi: 10.1111/j.1365-3059.2011.02472.x.

Shaner, G., and Finney, R. F. (1977). The effect of nitrogen fertilization on the expression of slow-mildewing resistance in knox wheat. *Phytopathology* 67, 1051–1056. doi: 10.1094/Phyto-67-105
